# Targeting DNA Damage Response and Replication Stress in Pancreatic Cancer

**DOI:** 10.1053/j.gastro.2020.09.043

**Published:** 2021-01

**Authors:** Stephan B. Dreyer, Rosie Upstill-Goddard, Viola Paulus-Hock, Clara Paris, Eirini-Maria Lampraki, Eloise Dray, Bryan Serrels, Giuseppina Caligiuri, Selma Rebus, Dennis Plenker, Zachary Galluzzo, Holly Brunton, Richard Cunningham, Mathias Tesson, Craig Nourse, Ulla-Maja Bailey, Marc Jones, Kim Moran-Jones, Derek W. Wright, Fraser Duthie, Karin Oien, Lisa Evers, Colin J. McKay, Grant A. McGregor, Aditi Gulati, Rachel Brough, Ilirjana Bajrami, Stephan Pettitt, Michele L. Dziubinski, Juliana Candido, Frances Balkwill, Simon T. Barry, Robert Grützmann, Lola Rahib, Glasgow Precision Oncology Laboratory,, Sarah Allison, Peter J. Bailey, Ulla-Maja Bailey, Andrew V. Biankin, Dario Beraldi, Holly Brunton, Giuseppina Caligiuri, Euan Cameron, David K. Chang, Susanna L. Cooke, Richard Cunningham, Stephan Dreyer, Paul Grimwood, Shane Kelly, Eirini-Maria Lampraki, John Marshall, Sancha Martin, Brian McDade, Daniel McElroy, Elizabeth A. Musgrove, Craig Nourse, Viola Paulus-Hock, Donna Ramsay, Rosie Upstill-Goddard, Derek Wright, Marc D. Jones, Lisa Evers, Selma Rebus, Lola Rahib, Bryan Serrels, Jane Hair, Nigel B. Jamieson, Colin J. McKay, Paul Westwood, Nicola Williams, Fraser Duthie, Australian Pancreatic Cancer Genome Initiative, Andrew V. Biankin, Amber L. Johns, Amanda Mawson, David K. Chang, Christopher J. Scarlett, Mary-Anne L. Brancato, Sarah J. Rowe, Skye H. Simpson, Mona Martyn-Smith, Michelle T. Thomas, Lorraine A. Chantrill, Venessa T. Chin, Angela Chou, Mark J. Cowley, Jeremy L. Humphris, Marc D. Jones, R. Scott Mead, Adnan M. Nagrial, Marina Pajic, Jessica Pettit, Mark Pinese, Ilse Rooman, Jianmin Wu, Jiang Tao, Renee DiPietro, Clare Watson, Angela Steinmann, Hong Ching Lee, Rachel Wong, Andreia V. Pinho, Marc Giry-Laterriere, Roger J. Daly, Elizabeth A. Musgrove, Robert L. Sutherland, Sean M. Grimmond, Nicola Waddell, Karin S. Kassahn, David K. Miller, Peter J. Wilson, Ann-Marie Patch, Sarah Song, Ivon Harliwong, Senel Idrisoglu, Craig Nourse, Ehsan Nourbakhsh, Suzanne Manning, Shivangi Wani, Milena Gongora, Matthew Anderson, Oliver Holmes, Conrad Leonard, Darrin Taylor, Scott Wood, Christina Xu, Katia Nones, J. Lynn Fink, Angelika Christ, Tim Bruxner, Nicole Cloonan, Felicity Newell, John V. Pearson, Peter Bailey, Michael Quinn, Shivashankar Nagaraj, Stephen Kazakoff, Nick Waddell, Keerthana Krisnan, Kelly Quek, David Wood, Jaswinder S. Samra, Anthony J. Gill, Nick Pavlakis, Alex Guminski, Christopher Toon, Ray Asghari, Neil D. Merrett, Darren Pavey, Amitabha Das, Peter H. Cosman, Kasim Ismail, Chelsie O’Connnor, Vincent W. Lam, Duncan McLeod, Henry C. Pleass, Arthur Richardson, Virginia James, James G. Kench, Caroline L. Cooper, David Joseph, Charbel Sandroussi, Michael Crawford, James Gallagher, Michael Texler, Cindy Forest, Andrew Laycock, Krishna P. Epari, Mo Ballal, David R. Fletcher, Sanjay Mukhedkar, Nigel A. Spry, Bastiaan DeBoer, Ming Chai, Nikolajs Zeps, Maria Beilin, Kynan Feeney, Nan Q. Nguyen, Andrew R. Ruszkiewicz, Chris Worthley, Chuan P. Tan, Tamara Debrencini, John Chen, Mark E. Brooke-Smith, Virginia Papangelis, Henry Tang, Andrew P. Barbour, Andrew D. Clouston, Patrick Martin, Thomas J. O’Rourke, Amy Chiang, Jonathan W. Fawcett, Kellee Slater, Shinn Yeung, Michael Hatzifotis, Peter Hodgkinson, Christopher Christophi, Mehrdad Nikfarjam, Angela Mountain, Victorian Cancer Biobank, James R. Eshleman, Ralph H. Hruban, Anirban Maitra, Christine A. Iacobuzio-Donahue, Richard D. Schulick, Christopher L. Wolfgang, Richard A. Morgan, Mary Hodgin, Aldo Scarpa, Rita T. Lawlor, Stefania Beghelli, Vincenzo Corbo, Maria Scardoni, Claudio Bassi, Margaret A. Tempero, Andrew V. Biankin, Sean M. Grimmond, David K. Chang, Elizabeth A. Musgrove, Marc D. Jones, Craig Nourse, Nigel B. Jamieson, Janet S. Graham, Andrew V. Biankin, David K. Chang, Nigel B. Jamieson, Janet S. Graham, Amber Johns, Marina Pajic, Fieke E.M. Froeling, Phillip Beer, Elizabeth A. Musgrove, Gloria M. Petersen, Alan Ashworth, Margaret C. Frame, Howard C. Crawford, Diane M. Simeone, Chris Lord, Debabrata Mukhopadhyay, Christian Pilarsky, David A. Tuveson, Susanna L. Cooke, Nigel B. Jamieson, Jennifer P. Morton, Owen J. Sansom, Peter J. Bailey, Andrew V. Biankin, David K. Chang

**Affiliations:** 1 Glasgow Precision Oncology Laboratory, University of Glasgow, Institute of Cancer Sciences, Wolfson Wohl Cancer Research Centre, Glasgow, United Kingdom; 2 West of Scotland Pancreatic Unit, Glasgow Royal Infirmary, Glasgow, United Kingdom; 3 Department of Pathology, Southern General Hospital, Greater Glasgow and Clyde National Health Service, Glasgow, United Kingdom; 4 West of Scotland Genetic Services, National Health Service, Greater Glasgow and Clyde, Queen Elizabeth University Hospital Campus, Glasgow, United Kingdom; 1 The Kinghorn Cancer Centre, Garvan Institute of Medical Research, 370 Victoria Street, Darlinghurst, Sydney, New South Wales, Australia; 2 Wolfson Wohl Cancer Research Centre, Institute of Cancer Sciences, University of Glasgow, Glasgow, Scotland, United Kingdom; 3 Queensland Centre for Medical Genomics, Institute for Molecular Bioscience, University of Queensland, St Lucia, Queensland, Australia; 4 Royal North Shore Hospital, St Leonards, New South Wales, Australia; 5 Bankstown Hospital, Bankstown, New South Wales, Australia; 6 Liverpool Hospital, Liverpool, New South Wales, Australia; 7 Westmead Hospital, Westmead, New South Wales, Australia; 8 Royal Prince Alfred Hospital, Camperdown, New South Wales, Australia; 9 Fremantle Hospital, Fremantle, Western Australia, Australia; 10 Sir Charles Gairdner Hospital, Nedlands, Western Australia, Australia; 11 St John of God Healthcare, Subiaco, Western Australia, Australia; 12 Royal Adelaide Hospital, Adelaide, South Australia, Australia; 13 Flinders Medical Centre, Bedford Park, South Australia, Australia; 14 Greenslopes Private Hospital, Greenslopes, Queensland, Australia; 15 Envoi Pathology, Herston, Queensland, Australia; 16 Princess Alexandria Hospital, Woolloongabba, Queensland, Australia; 17 Austin Hospital, Heidelberg, Victoria, Australia; 18 Victorian Cancer Biobank, Carlton, Victoria, Australia; 19 Johns Hopkins Medical Institute, Baltimore, Maryland; 20 ARC-NET Center for Applied Research on Cancer, University of Verona, Verona, Italy; 21 University of California, San Francisco, San Francisco, California; 22 Greater Glasgow and Clyde National Health Service, Glasgow, United Kingdom; 1 Wolfson Wohl Cancer Research Centre, Institute of Cancer Sciences, University of Glasgow, Glasgow, Scotland, United Kingdom; 2 West of Scotland Pancreatic Unit, Glasgow Royal Infirmary, Glasgow, United Kingdom; 3 Cancer Research UK Beatson Institute, Glasgow, United Kingdom; 4 Department of Pharmacological Faculty, Université Grenoble Alpes, Saint-Martin-d’Heres, France; 5 Department of Biochemistry and Structural Biology, University of Texas Health San Antonio, San Antonio, Texas; 6 Medical Research Council Institute of Genetics and Molecular Medicine, Edinburgh Cancer Research UK Centre, University of Edinburgh, Edinburgh, United Kingdom; 7 Cold Spring Harbor Laboratory, Cold Spring Harbor, New York; 8 Lustgarten Foundation Pancreatic Cancer Research Laboratory, Cold Spring Harbor, New York; 9 Stratified Medicine Scotland, Queen Elizabeth University Hospital, Glasgow, United Kingdom; 10 College of Medicine, Veterinary, and Life Sciences, University of Glasgow, Glasgow, United Kingdom; 11 Department of Pathology, Queen Elizabeth University Hospital, Glasgow, United Kingdom; 12 Greater Glasgow and Clyde Bio-repository, Pathology Department, Queen Elizabeth University Hospital, Glasgow, United Kingdom; 13 Cancer Research UK Gene Function Laboratory and Breast Cancer Now Toby Robins Research Centre, The Institute of Cancer Research, London, United Kingdom; 14 Department of Molecular and Integrative Physiology, University of Michigan, Ann Arbor, Michigan; 15 Barts Cancer Institute, Queen Mary University of London, London, United Kingdom; 16 Bioscience, Oncology, Innovative Medicines and Early Development Biotech Unit, AstraZeneca, Cambridge, United Kingdom; 17 Department of Surgery, Universitätsklinikum Erlangen, Erlangen, Germany; 18 Pancreatic Cancer Action Network, Manhattan Beach, California; 19 Glasgow Precision Oncology Laboratory, Glasgow, United Kingdom; 20 Australian Pancreas Genome, Darlinghurst, Australia; 21 The Kinghorn Cancer Centre, Darlinghurst and Garvan Institute of Medical Research, Sydney, Australia; 22 Epigenetics Unit, Department of Surgery and Cancer, Imperial College London, Hammersmith Campus, London, United Kingdom; 23 Sanger Institute, Wellcome Genome Campus, Cambridge, United Kingdom; 24 Mayo Clinic, Rochester, Minnesota; 25 University of California–San Francisco Helen Diller Family Comprehensive Cancer Center, San Francisco, California; 26 Pancreatic Cancer Center, Perlmutter Cancer Center, New York University Langone Health, New York, New York; 27 Department of Biochemistry and Molecular Biology, Mayo Clinic College of Medicine and Science, Jacksonville, Florida; 28 South Western Sydney Clinical School, Faculty of Medicine, University of New South Wales, Liverpool, Australia

**Keywords:** Pancreatic Cancer, DNA Damage Response, Replication Stress, Personalized Medicine, DDR, DNA damage response, DMSO, dimethyl sulfoxide, EC_50_, median effective concentration, GO, Gene Ontology, HR, homologous recombination, HRD, homologous recombination deficiency, ICGC, International Cancer Genome Consortium, MTS, 3-(4,5-dimethylthiazol-2-yl)-5-(3-carboxymethoxyphenyl)-2-(4-sulfophenyl)-2H-tetrazolium, PC, pancreatic cancer, PDAC, pancreatic ductal adenocarcinoma, PDCL, patient-derived cell line, PDX, patient-derived xenograft, RNAseq, RNA sequencing, RPPA, reverse-phase protein array, siRNA, small interfering RNA, SV, structural variation, TOM, topological overlap measure

## Abstract

**Background & Aims:**

Continuing recalcitrance to therapy cements pancreatic cancer (PC) as the most lethal malignancy, which is set to become the second leading cause of cancer death in our society. The study aim was to investigate the association between DNA damage response (DDR), replication stress, and novel therapeutic response in PC to develop a biomarker-driven therapeutic strategy targeting DDR and replication stress in PC.

**Methods:**

We interrogated the transcriptome, genome, proteome, and functional characteristics of 61 novel PC patient–derived cell lines to define novel therapeutic strategies targeting DDR and replication stress. Validation was done in patient-derived xenografts and human PC organoids.

**Results:**

Patient-derived cell lines faithfully recapitulate the epithelial component of pancreatic tumors, including previously described molecular subtypes. Biomarkers of DDR deficiency, including a novel signature of homologous recombination deficiency, cosegregates with response to platinum (*P* < .001) and PARP inhibitor therapy (*P* < .001) in vitro and in vivo. We generated a novel signature of replication stress that predicts response to ATR (*P* < .018) and WEE1 inhibitor (*P* < .029) treatment in both cell lines and human PC organoids. Replication stress was enriched in the squamous subtype of PC (*P* < .001) but was not associated with DDR deficiency.

**Conclusions:**

Replication stress and DDR deficiency are independent of each other, creating opportunities for therapy in DDR-proficient PC and after platinum therapy.

## Graphical abstract

**Figure:**
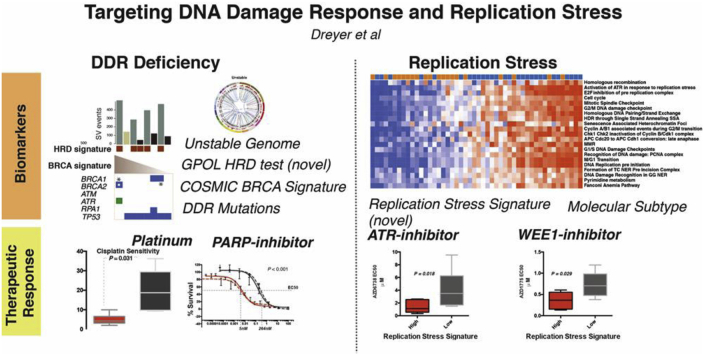

**What You Need to Know**

**Background and Context**

Pancreatic cancer (PC) remains a highly lethal malignancy with few successful therapeutic options. There is a growing list of novel agents that target DNA replication and repair, which may offer treatment options for patients with PC.

**New Findings**

A subset of PC demonstrate evidence of high replication stress. This is enriched in the squamous transcriptomic subtype of PC. A novel transcriptomic signature of replication stress predicts response to novel ATR and WEE1 inhibitors. High replication stress and DNA damage response (DDR) deficiency are separate entities that can exist independently and be targeted with different agents.

**Limitations**

This study is limited that the novel therapeutic data is based on preclinical models of patient derived cell lines and organoid responses. Human PC response data is not yet available, as the ATR and WEE1 inhibitors are at early stages of therapeutic development in PC.

**Impact**

High replication stress and DDR deficiency exist independently of each other, offering therapeutic opportunities in DDR proficient PC with high replication stress using ATR or WEE1 inhibitors. It also provides a potential therapeutic strategy in the acquired platinum resistance setting.

Pancreatic cancer (PC) has recently overtaken breast cancer to become the third leading cause of cancer death in the United States,^1^ and it is predicted to become the second within a decade.^2^ Pancreatic ductal adenocarcinoma (PDAC), the more common form of PC, is dominated by mutations in 4 well-known cancer genes (*KRAS*, *TP53*, *CDKN2A*, and *SMAD4*). Only a few genes are mutated in 5%–15% of cases, amidst an ocean of infrequently mutated genes in the majority of patients.^3^, ^4^, ^5^, ^6^, ^7^, ^8^, ^9^, ^10^, ^11^ This diversity may explain the lack of progress with targeted therapies, because actionable genomic events being targeted therapeutically are present in only a small proportion of unselected participants in clinical trials.^12^ To better select patients to clinical trials, biomarkers that predict response to novel and established treatments are urgently needed and must extend beyond the detection of point mutations in coding genes and low-prevalence actionable genomic events.

Although molecular subtyping of cancer based on biological attributes can facilitate drug discovery, to be clinically relevant, the optimal taxonomy must inform patient management through prognostication or, more importantly, treatment selection.^13^ Recent studies have subtyped PC in various ways,^5^^,^^9^^,^^14^, ^15^, ^16^, ^17^, ^18^ grouping similarities based on structural attributes of genomes, genes mutated in pathways, or molecular mechanisms inferred through messenger RNA expression. We recently defined 4 transcriptomic subtypes of PC,^5^^,^^19^ with 2 distinct primary lineages, termed *classical pancreatic* (which can be further divided into pancreatic progenitor, immunogenic, and aberrantly differentiated endocrine exocrine subtypes) and *squamous*.^5^ Despite discrepancies in nomenclature, 1 molecular class (variably termed *quasi-mesenchymal*, *basal-like*, or *squamous*) is consistently defined and is associated with a poor prognosis.^19^^,^^20^ A key distinction is the epigenetic profile of the squamous subtype, with chromatin modification and methylation orchestrating the loss of pancreatic endodermal transcriptional networks and, as a consequence, suppressing transcripts that designate a pancreatic identity.^5^ These biologically based molecular taxonomies of PC, although associated with differences in outcome, have yet to inform treatment decisions.

DNA damage response (DDR) deficiency is a hallmark of cancer, including PC,^8^ and is thought to render some tumors preferentially sensitive to DNA-damaging agents such as platinum and PARP inhibitors. There is a growing compendium of novel therapeutics that target DNA damage response mechanisms and the cell cycle, such as ATR and WEE1 inhibitors.^21^ Genomic instability, a key feature of many cancers, typically secondary to defects in DNA replication and repair during the cell cycle, often results in replication stress.^22^^,^^23^ Oncogene activation drives replication stress, particularly through RAS and MYC signaling, both of which are prevalent molecular features of PC.^22^^,^^24^^,^^25^ The platinum-containing regimen, FOLFIRINOX, has become the standard of care for all stages of PC, yet it is suitable only for patients with good performance status; however, the majority of patients unfortunately do not respond.^26^, ^27^, ^28^ Consequently, many patients experience the morbidity, and even mortality, of systemic platinum chemotherapy with little or no survival benefit or quality of life. Biomarker-driven patient selection strategies and novel therapeutics that build on platinum response or disease stabilization that target DDR mechanisms provide a substantial opportunity to improve outcomes.

Building on previous work on DDR mechanisms and PC, we aim to expand the indications for novel DDR inhibitors beyond patients with defects in homologous recombination (HR) mechanisms. We aim to refine proposed DDR biomarkers of platinum response to be tested in prospective clinical trials and to correlate and overlap this with cell cycle inhibitor response to identify patients who will respond to novel agents, such as ATR and WEE1 inhibitors.

Here, we used 61 patient-derived cell lines (PDCLs) of PC (Supplementary Tables 1 and 2) to define subtype-specific molecular mechanisms and identify opportunities for molecular subtype–directed treatment selection that targets DDR mechanisms. We performed messenger RNA expression analysis (RNA sequencing [RNAseq]) (n = 48) complemented by whole genome sequencing (n = 47), which was further enhanced with reverse phase protein arrays (RPPAs), functional screenings using small interfering RNA (siRNA) and targeted functional analysis. To our knowledge, we identify novel biomarkers of DDR deficiency and replication stress with potential clinical utility that associate with therapeutic sensitivity. We show that DDR deficiency exists independently of replication stress, the previously identified poor-prognostic squamous subtype is enriched for replication stress, and transcriptomic readouts of replication stress confer sensitivity to therapeutics that target the cell cycle checkpoint machinery.

## Materials and Methods

The full methods and additional references can be found in the Supplementary Material.

### Human Research Ethics Approvals

Ethical approval was obtained for all human samples and data (Supplementary Materials).

### Cell Culture

PDCLs were generated as previously described.^4^^,^^29^, ^30^, ^31^ PDCLs were cultured in conditions specifically formulated for each individual line based on growth preferences and those resulting in cell lines that most closely resembled physiologic cells from the initial tumor. Cells were grown in a humidified environment with either 5% or 2% CO_2_ at 37°C. All cell lines were profiled by short tandem repeat DNA profiling as unique (CellBankaustralia.com). Cell lines were tested routinely for mycoplasma contamination by using the MycoAlert PLUS Mycoplasma Detection Kit (Lonza, Basel, Switzerland; LT07–318).

### In Vitro Cytotoxicity Assays

Cells were seeded on 96-well plates (Costar, Corning, Corning, New York) and allowed to adhere for 24 hours. Cells were treated with increasing doses of cisplatin (Accord Healthcare, London, UK), AZD6738 (AstraZeneca, Cambridge, UK), AZD1775 (AstraZeneca), and AZD7762 (AstraZeneca) for 72 hours. Cells were treated with BMN-673 (Pfizer, New York, NY), Rucaparib (Clovis Oncology, Boulder, CO), CFI-400945 (Cayman Chemical, Ann Arbor, MI), and Palbociclib (Pfizer) for a total of 9 days, with repeated dosing every 72 hours in conjunction with changing cell media. Actinomycin D (Sigma-Aldrich, St Louis, MO), drug vehicle (dimethyl sulfoxide [DMSO]), and media-only controls were performed on each individual plate. For all other cytotoxicity assays, cells were plated in 96-well plates and treated with serial dilutions of indicated inhibitors 24 hours after plating for the indicated timepoints. Cell viability was determined by using the CellTiter 96 Aqueous nonradioactive cell proliferation assay (PROMEGA, United Kingdom) composed of solutions of a tetrazolium compound (3-[4,5-dimethylthiazol-2-yl]-5-[3-carboxymethoxyphenyl]-2-[4-sulfophenyl]-2H-tetrazolium, inner salt; [MTS]) and an electron-coupling reagent (phenazine methosulfate) (Promega, Madison, WI). The assay was performed at an absorbance of 490 nm using an enzyme-linked immunosorbent assay plate reader (Tecan Trading AG, Männedorf, Germany). Background absorbance was corrected for by wells containing medium alone, and the absorbance was normalized to a scale of 0% (complete cell death by actinomycin D (5–10 μg/mL) to 100% (no drug). At least 3 biological repeats were performed for each experiment. Median effective concentration calculation and dose response curves were generated with GraphPad Prism 6 (GraphPad Software, La Jolla, CA).

### Organoid Drug Screening

Therapeutic sensitivity in the organoids was assessed as previously described.^32^ Organoids were dissociated into single cells. One thousand viable cells were plated per well in 20 μL 10% Matrigel/human complete organoid media (Corning Life Sciences, United Kingdom). Increasing concentrations of AZD6738 (AstraZeneca) and AZD1775 (AstraZeneca) were added 24 hours after plating, after the reformation of organoids was visually verified. Compounds were dissolved in DMSO, and all treatment wells were normalized to 0.5% DMSO content. After 7 days, cell viability was assessed by using CellTiter-Glo (Promega) as per the manufacturer’s instructions on a SpectraMax I3 (Molecular Devices, San Jose, CA) plate reader. At least 3 biological repeats were performed for each experiment. Median inhibitory concentration calculation and dose response curves were generated with GraphPad Prism 6.

### Patient-Derived Xenografts

Patient-derived xenografts (PDXs) of PDAC were generated and comprehensively characterized as part of the International Cancer Genome Consortium (ICGC) project. BALB/c nude mice were anesthetized, and a single PDX fragment was inserted subcutaneously into the right flank according to standard operating procedure. PDX models were grown to 150 mm^3^ (volume = [length^2^ × width]/2); at this point, each PDX was randomized to a different treatment regimen. Responsive PDXs were treated once tumor size returned to 150 mm^3^, up to a maximum of 3 rounds. Resistant models were treated after a treatment break of 2 weeks in accordance with current clinical treatment regimes, up to a maximum of 2 rounds. Each experiment was terminated once tumor volume reached the endpoint (750 mm^3^), in accordance with home office animal welfare regulations. Full methods can be found in Supplementary Materials.

### γH2AX and pRPA Foci Formation Assay

PDCLs were cultured as standard and seeded in 96-well plates at a concentration of 10^4^ cells per well. At 24 hours after seeding, cells were either left untreated or exposed to 4 Gy of ionizing radiation and processed for analysis at 2, 4, and 20 hours after exposure. Cells were stained with primary antibodies at a dilution of 1:1000 with anti-pRPA32 (S4/S8; Bethyl Laboratories, Montgomery, TX) and anti-γH2AX (Ser139; Merck, Kenilworth, NJ). Secondary antibodies used were Alexa 488 anti-mouse IgG (green) and Cy3 anti-rabbit IgG (Sigma-Aldrich). 4′,6-Diamidino-2-phenylindole (Life Technologies, Rockville, MD) was used as a nuclear stain. Confocal imaging was performed by using the Opera Phenix high-content screening system (PerkinElmer, Waltham, MA) at ×63 magnification using a water objective, at wavelengths of 405 nm (4′,6-diamidino-2-phenylindole), 488 nm (Alexa), and 561 nm (Cy3). A minimum of 320 cells (median, 980; range, 322–1886), in 2 separate experiments, were analyzed for each time point. Image analysis was performed by using the Columbus Image data storage and analysis system (PerkinElmer). Statistical analysis was performed with GraphPad Prism 6.

### Nucleic Acid Extraction

DNA and RNA extraction were performed by using previously published methods.^4^

### Whole-Genome Library Preparation

Whole-genome libraries were generated by using either the Illumina (San Diego, CA) TruSeq DNA LT sample preparation kit (part nos. FC-121–2001 and FC-121–2001) or the Illumina TruSeq DNA PCR-Free LT sample preparation kit (Illumina, part nos. FC-121–3001 and FC-121–3002) according to the manufacturer’s protocols with some modifications (Illumina, part no. 15026486 Rev. C July 2012 and 15036187 Rev. A January 2013 for the 2 different kits, respectively). Full methods can be found in the Supplementary Materials.

### RNA Sequencing Library Generation and Sequencing

RNA-seq libraries were generated by using the Illumina TruSeq Stranded Total RNA (part no. 15031048 Rev. D April 2013) kits on a Perkin Elmer Sciclone G3 NGS Workstation (product no. SG3-31020-0300). Full methods can be found in the Supplementary Material.

### Library Sequencing

All libraries were sequenced by using the Illumina HiSeq 2000/2500 system with TruSeq SBS Kit v3-HS (200 cycles) reagents (part no. FC-401-3001) to generate paired-end 101–base pair reads.

### Copy Number Analysis

Matched tumor and normal patient DNA was assayed by using Illumina SNP BeadChips as per manufacturer’s instructions (HumanOmni1-Quad or HumanOmni2.5–8 BeadChips) and analyzed as previously described.

### Identification and Verification of Structural Variants

The somatic structural variant pipeline was identified by using the qSV tool. A detailed description of its use has been recently published.^4^^,^^33^

### Identification of and Verification of Point Mutations

Substitutions and insertions/deletions were called by using a consensus calling approach that included qSNP, GATK, and Pindel. The details of call integration and filtering and verification using orthogonal sequencing and matched sample approaches are as previously described.^4^^,^^33^^,^^34^

### Mutational Signatures

Mutational signatures were defined for genome-wide somatic substitutions, as previously described.^4^

### HRDetect

HRDetect scores were calculated as previously described for the PDCLs.^35^

### Small Interfering RNA Screening and Analysis

Full siRNA screening and analysis were performed with established methods and are described in the Supplementary Material.

### Reverse-Phase Protein Array

RPPA was performed to investigate proteomic differences between PDCLs by using established methods and is fully described in the Supplementary Materials.

### RNA-Sequencing Analysis

RNA-seq read mapping was performed by using the bcbio-nextgen project RNAseq pipeline (https://bcbio-nextgen.readthedocs.org/en/latest/). Briefly, after quality control and adaptor trimming, reads were aligned to the GRCh37 genome build by using STAR counts for known genes, which were generated with the function featureCounts in the R (R Foundation for Statistical Computing, Vienna, Austria)/Bioconductor package Rsubread. The R/Bioconductor package DESeq2 was used to normalize count data among samples and to identify differentially expressed genes. Expression data were normalized by using the rlog transform in the DESeq2 package, and these values were used for all downstream analyses.

### Weighted Gene Coexpression Network Analysis

Weighted gene coexpression network analysis was used to generate a transcriptional network from rlog-normalized RNAseq data. Briefly, weighted gene coexpression network analysis clusters genes into network modules by using a topological overlap measure (TOM). The TOM is a highly robust measure of network interconnectedness and essentially provides a measure of the connection strength between 2 adjacent genes and all other genes in a network. Genes are clustered by using 1 – TOM as the distance measure, and gene modules are defined as branches of the resulting cluster tree using a dynamic branch-cutting algorithm. Full methods can be found in the Supplementary Materials.

### Identification of Significant Subtype-Specific Changes in Pathways and/or Processes

The R package clipper was used to identify pathways and/or processes showing significant change between PDCL subtypes. clipper implements a 2-step empirical approach, using a statistical analysis of means and concentration matrices of graphs derived from pathway topologies, to identify signal paths having the greatest association with a specific phenotype.

### Pathway Analysis

Ontology and pathway enrichment analysis was performed by using the R package dnet and/or the ClueGO/CluePedia Cytoscape plugins, as indicated. Visualization and/or generation of network diagrams was performed using either Cytoscape or the R package RedeR.

### Glasgow Precision Oncology Laboratory Homologous Recombination Deficiency Test

Signature generation was done by using whole-genome sequencing data as previously described.^36^^,^^37^ A positive test was defined when the following criteria were met.

1. There were more than 50 structural variants in total.
2. Greater than 70% of the structural variants were deletions, duplications, or translocations.
3. The structural variation (SV) pattern was not focal, as defined by a large number of structural variants due to chromothripsis or amplifications.
4. If the predominant variant types were deletions and translocations, the median deletion size was <10 kilo base pairs.

OR

5. If the predominant variant type was duplication, the median duplication size was <50 kilo base pairs.

### Replication Stress Signature Generation

Differentially expressed genes were compared to genes associated with Gene Ontology (GO) terms using the R package dnet. Significantly expressed GO terms involved in DNA damage response and cell cycle control were selected. Differential expression of each selected GO term was applied to each individual PDCL and patient derived organoid that underwent RNAseq. This, in turn, was used to generate a composite score by totaling the score for each selected GO term sig.score. The function from the R package genefu was used to calculate a specific signature score in a given sample using the signatures generated for each pathway and/or process. This was termed the *replication stress signature*. Generation of the signature score for bulk tumor samples followed the same methodology.

### Bulk Expression Sets and Immune Signature Scores

Bulk RNAseq expression data, subtype assignments, and immune signature scores were obtained from Bailey et al.^5^

### Gene Set Enrichment of Pancreatic Ductal Adenocarcinoma Subtypes

Gene set enrichment was performed by using the R package GSVA. Gene sets representing PDAC subtypes were generated as previously described.

### Clustering and Subtype Assignment

The package ConsensusClusterPlus was used to classify PDCLs according to the expression signatures defined by Moffitt et al^18^ and Bailey et al.^5^ Gene sets representing PDAC subtypes were generated as previously described.

### Statistical Analysis

A Kruskal-Wallis test was applied to the indicated stratified scores to determine whether distributions were significantly different. Fisher exact tests were used to evaluate the association between dichotomous variables.

### Plot Generation

Heatmaps and oncoplots were generated by using the R package ComplexHeatmap*.* Dot charts, density plots, and boxplots were generated using the R package ggpubr*.* Violin plots were generated with the Python package Seaborn*.* Biplot was generated with the R package ggfortify. All other plots were generated with the R package ggplot2.

## Results

### Patient-Derived Cell Lines Recapitulate Pancreatic Cancer Subtypes

**Figure 1:**
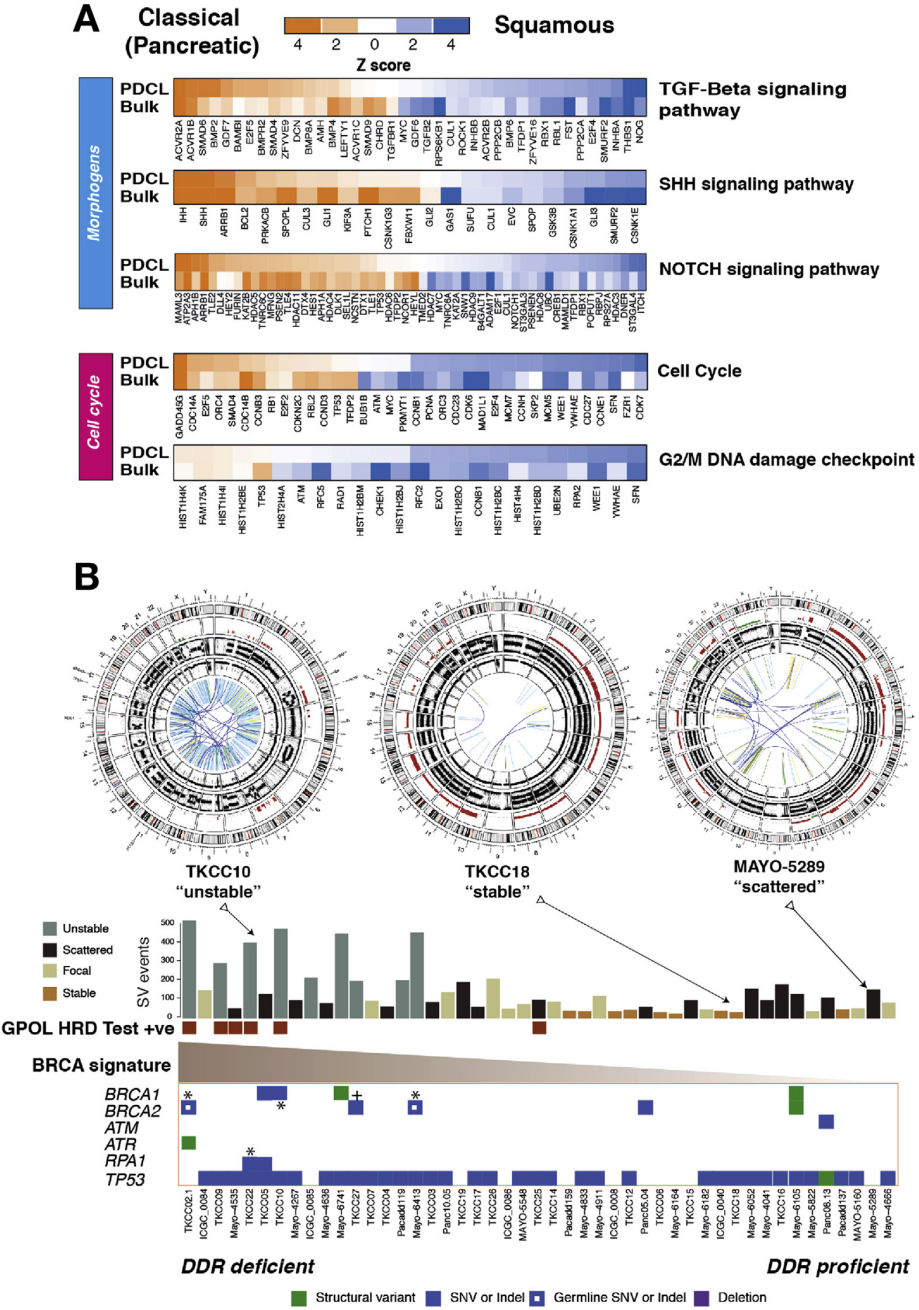
Subtype specific differences and DDR in PDCLs of PC. (*A*) Heatmaps of key genes in pathways important in carcinogenesis, grouped into distinct molecular processes related to morphogenesis and cell cycle control between molecular subtypes of bulk tumor and PDCLs of PC. The degree of color saturation is proportional to the degree of enrichment in the squamous (*blue*) and classical pancreatic (*orange*) subtypes. For all samples within each subtype, genes are ranked by the most differentially expressed between subtypes. (*B*) Surrogate biomarkers of DDR deficiency, defined by large-scale sequencing projects of PC, include (1) unstable genome (>200 SVs), (2) the novel GPOL HRD test, (3) high-ranking BRCA mutational signature, and (4) deleterious mutations in DDR pathway genes. PDCLs are ranked from left to right based on the COSMIC BRCA mutational signature, with SV subtype, number of SVs, and GPOL HRD test status symbolized on the top bar. Examples of circos plots for 3 PDCLs are included, representing unstable, stable, and scattered subtypes. SNV, single-nucleotide variant; TGF, transforming growth factor.

**Supplementary Figure 1:**
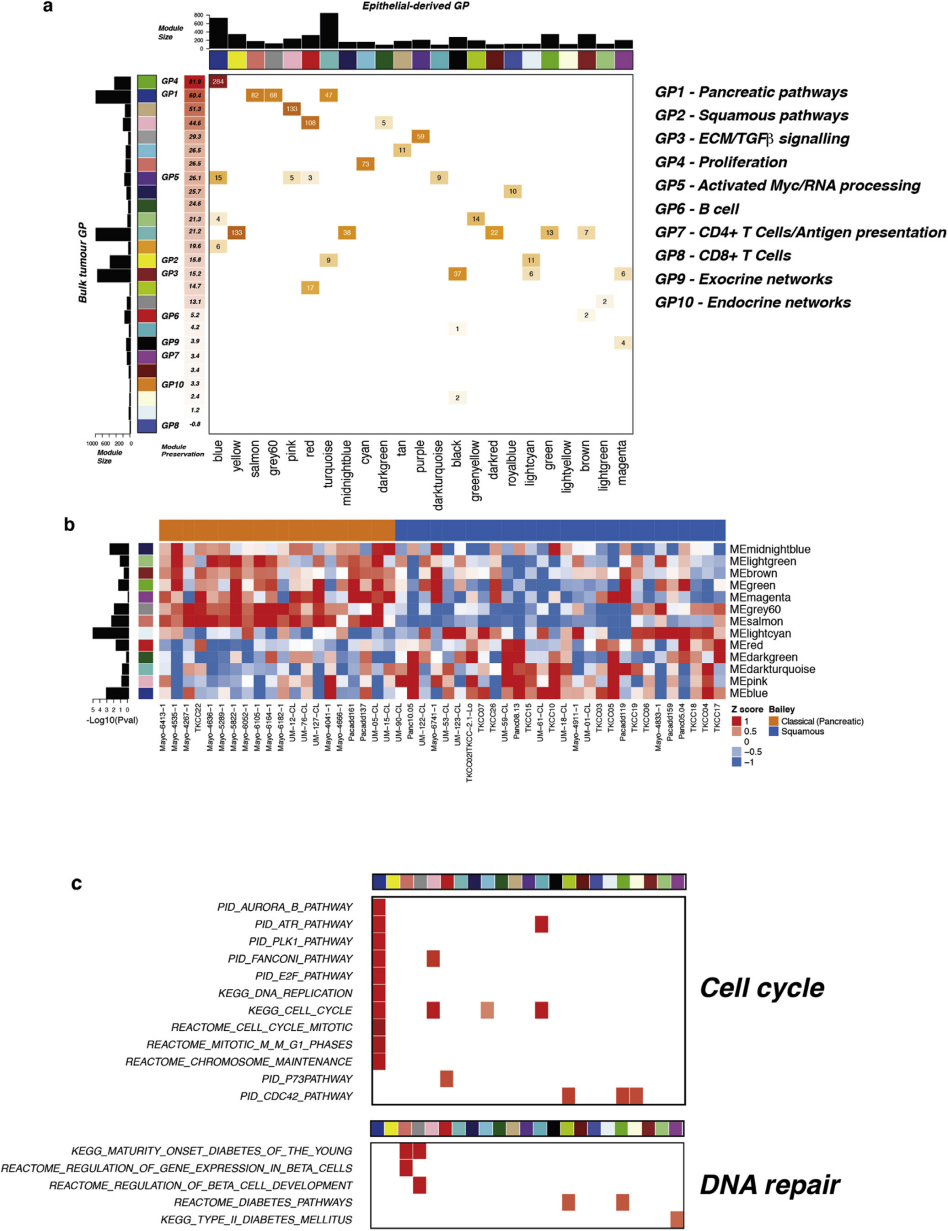
Gene program dysregulation in PC PDCLs. (*A*) Comparison of gene programs (GP) of bulk tumor previously described^28^ and GPs identified in PDCLs by WGCNA; ranked by GP module preservation on the *x*-axis. (*B*) Heatmap of PDCLs classified into squamous (*blue*) and classical pancreatic (*orange*) subtypes showing GP module eigengene (ME) values. (*C*) The expression of components of publicly curated molecular pathways and mechanisms in PDCLs related to replication and DNA damage repair. GPs are grouped into key molecular processes that are important in carcinogenesis. ECM, extracellular matrix; TGF, transforming growth factor.

Hierarchical clustering of RNAseq data from the 48 PDCLs recapitulated the 2 primary classes of PC (Figure 1*A*, Supplementary Figure 1, and Supplementary Tables 3-5). Twenty-eight (58%) of the PDCLs were classified as squamous, and 20 (42%) were classical (Supplementary Table 4). The preservation of the 26 transcriptional networks (Gene Programs) we previously described in bulk PC^5^ was compared to PDCL-derived gene programs (Supplementary Figure 1 and Supplementary Tables 6 and 7). In total, 17 of the 26 gene programs were closely recapitulated in the PDCLs (Supplementary Figure 1), with the expected absence of immune infiltrate–related transcriptional networks (Supplementary Table 7)*.* The lack of stroma permitted higher resolution of epithelial transcriptomic networks, showing key mechanisms that are difficult to discern from biopsy samples. Differential expression of genes related to DNA damage response, cell cycle control, and morphogenic processes were observed between subtypes and correlated in both PDCLs and bulk tumor samples (Figure 1*A*). These findings suggest that PDCLs are representative of bulk PC and can be used to develop novel DDR therapeutic strategies for the clinic and that epithelial cell purity can provide greater sensitivity in detecting aberrant mechanisms.

### Biomarkers of DNA Damage Response Deficiency in Pancreatic Ductal Adenocarcinoma of Pancreatic Cancer

**Supplementary Figure 2:**
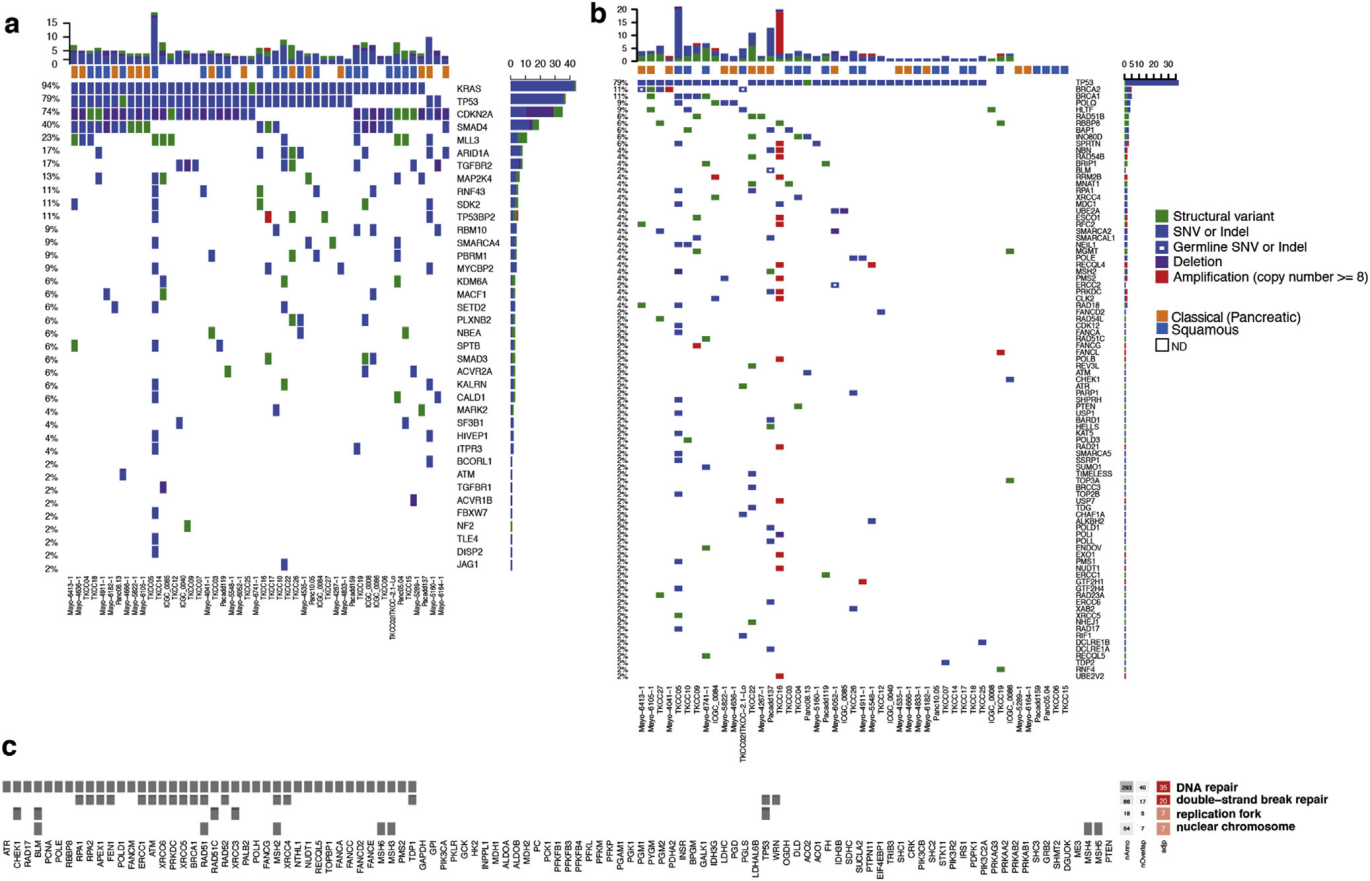
Mutational landscape and subtype-specific siRNA screening dependencies of PC PDCLs. (*A*) Oncoplot of somatic mutations of significantly mutated genes in PDCLs. Structural variations (*green*), nonsilent mutations (*blue*), deletion (*purple*), and amplification of ≥8 copies (*red*). (*B*) Oncoplot of somatic and germline mutations in key genes known to contribute to DDR and its mutation rate. (*C*) siRNA hits across all PDCLs grouped into molecular processes of DDR.

Various biomarkers of DDR deficiency associated with therapeutic response have been proposed but not validated clinically in PC. High-ranking Catalogue of Somatic Mutations in Cancer (COSMIC) Breast Cancer gene (BRCA) point mutational signature cosegregates with a high prevalence of structural variants, termed the *unstable* genomic subtype, and deleterious mutations in HR repair pathway genes such as *BRCA1* and *2* and *PALB2*^4^ (Figure 1*B* and Supplementary Tables 8-10). We previously showed that these signatures are associated with retrospective clinical response to platinum in PC.^4^ More recently, early data also suggest a therapeutic signal using PARP inhibitors; however, efficacy is not well defined beyond *BRCA1*/*2* mutations.^36^^,^^37^ To address this, we defined PDCLs as DDR deficient based on the presence of any of 4 putative biomarkers: (1) SV number and pattern (>200 SVs = unstable genome), (2) a high COSMIC BRCA mutational signature (ranked within top quintile), (3) a positive GPOL HR deficiency (HRD) test,^38^^,^^39^ and (4) mutations in key DDR genes (*BRCA1*, *BRCA2*, *ATM*, *ATR*, *RPA1*, *RAD51*, *RAD54*, and *FANCA*) (Figure 1*B*, Supplementary Figure 2, and Supplementary Table 11). Out of 47 PDCLs with whole-genome sequencing data, 6 (13%) had a positive GPOL HRD test result; 9 (19%) had >200 SVs (unstable genome); and 10 (21%) had mutations in DDR genes, of which 2 were germline variants (both in *BRCA2*) (Figure 1*B*, and Supplementary Table 12). There were 4 PDCLs with homozygous mutations in either *BRCA1*, *BRCA2*, or *RPA1*; these were all associated with unstable genomes, and 3 of these were positive on GPOL HRD test (Figure 1*B* and )Supplementary Table 3. Significant overlap existed among these, with n = 3 PDCLs with all 4 biomarkers present; n = 1 had 3 (unstable genome, GPOL HRD test, BRCA mutational signature) biomarkers positive, n = 4 were positive for 2 biomarkers, and the remaining n = 8 had 1 biomarker positive (Supplementary Table 13). There was no association between transcriptomic subtype and DDR status (*P* = .706).

### DNA Damage Response Deficiency Cosegregates With Response to Platinum and PARP Inhibitor Treatment

**Figure 2:**
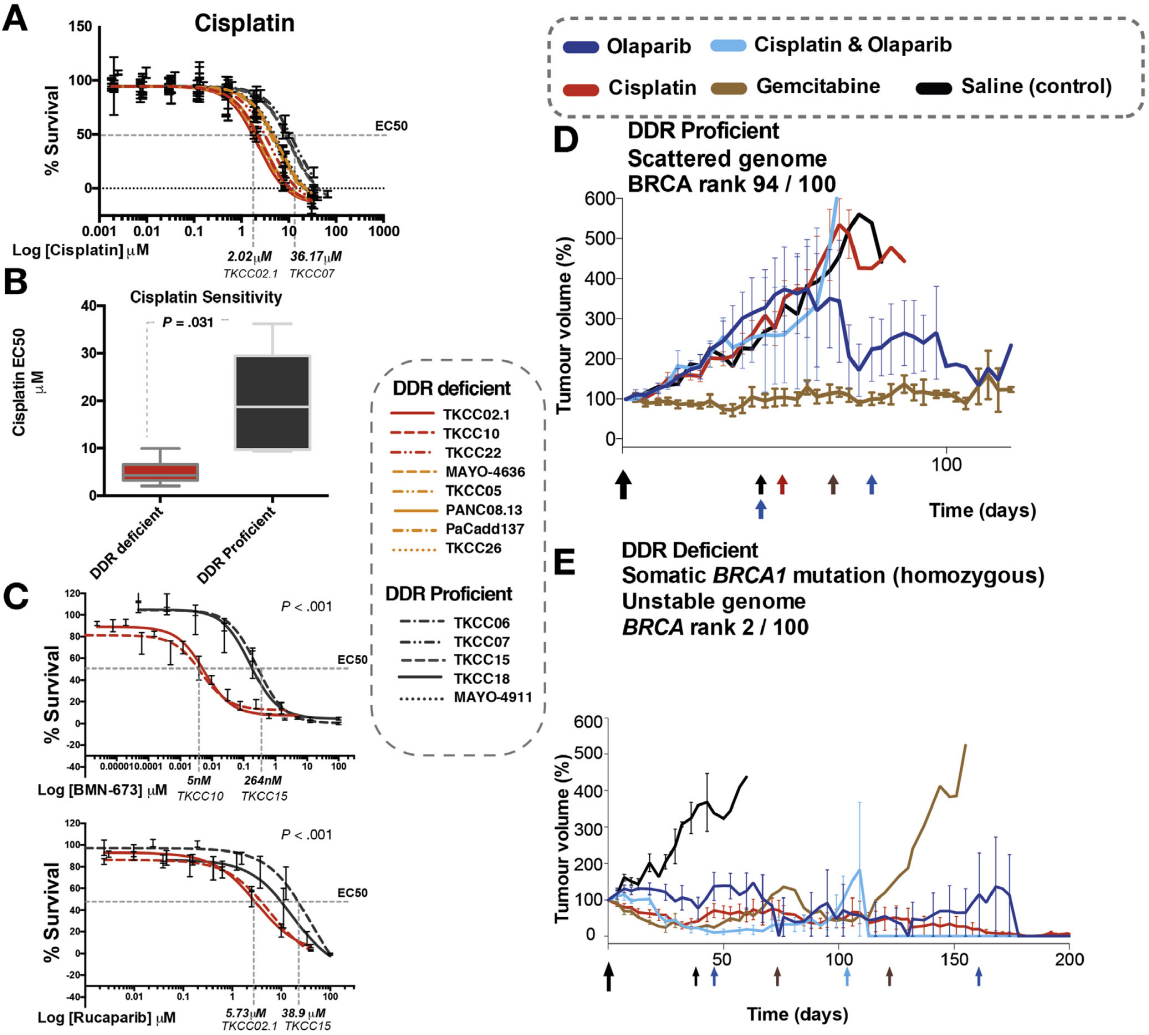
Targeting DDR-deficient PC with platinum and PARP inhibitors. (*A*) Cell viability after 72 hours of cisplatin treatment in PDCLs. The dotted line indicates that EC_50_ in the most sensitive PDCL was approximately 15 times more sensitive than the most resistant PDCLs. (*B*) Boxplot of mean cisplatin EC_50_ in PDCLS stratified by DDR status. The boxes represent the 95% confidence interval, and whiskers show the minimum and maximum range. *P* was calculated by using the Mann-Whitney test between the mean EC_50_ in each group. (*C*) PARP inhibitor (BMN-637 and rucaparib) response in PDCLs. The dotted lines indicate the EC_50_ between the most sensitive and most resistant PDCLs. *P* indicates the statistical difference between TKCC 10 (GPOL HRD test positive) and TKCC15 (DDR proficient) using nonlinear regression analysis. (*D*) PDX 2133 and (*E*) PDX 2179 (DDR deficient) treated with a panel of DNA-damaging agents and gemcitabine. The colored arrows indicate redosing of specific agents. M, mol/L.

To investigate the relationship between these putative biomarkers of DDR deficiency and platinum and PARP inhibitor response, cell viability assays were performed on 15 PDCLs. PDCLs defined as DDR deficient were more sensitive to both cisplatin therapy (*P* = .031) and PARP inhibition (*P* < .001) compared to DDR-proficient PDCLs (Figure 2 and Supplementary Table 11). The DDR-deficient PDCLs all had median effective concentrations (EC_50_s) to platinum of below the sensitivity threshold (10 μmol/L) set by large-scale pancancer cell line drug screenings (n = 880) using cisplatin (cancerrxgene.org [COSMIC]).

To further define clinically applicable therapeutic response biomarkers of DDR deficiency in vivo, bulk tumor PDX models that represent both DDR-proficient (PDX 2133) and -deficient (PDX 2179) PC were generated in balb/c nude mice. The DDR-proficient PDX did not respond to DNA-damaging agents, including cisplatin and the PARP inhibitor olaparib combination (Figure 2). The DDR-deficient PDX model, with a biallelic somatic loss-of-function *BRCA1* mutation, responded exceptionally to cisplatin and olaparib as monotherapy and in combination (Figure 2), suggesting that PARP inhibition can be as effective as platinum chemotherapy in DDR-deficient PC.

**Supplementary Figure 3:**
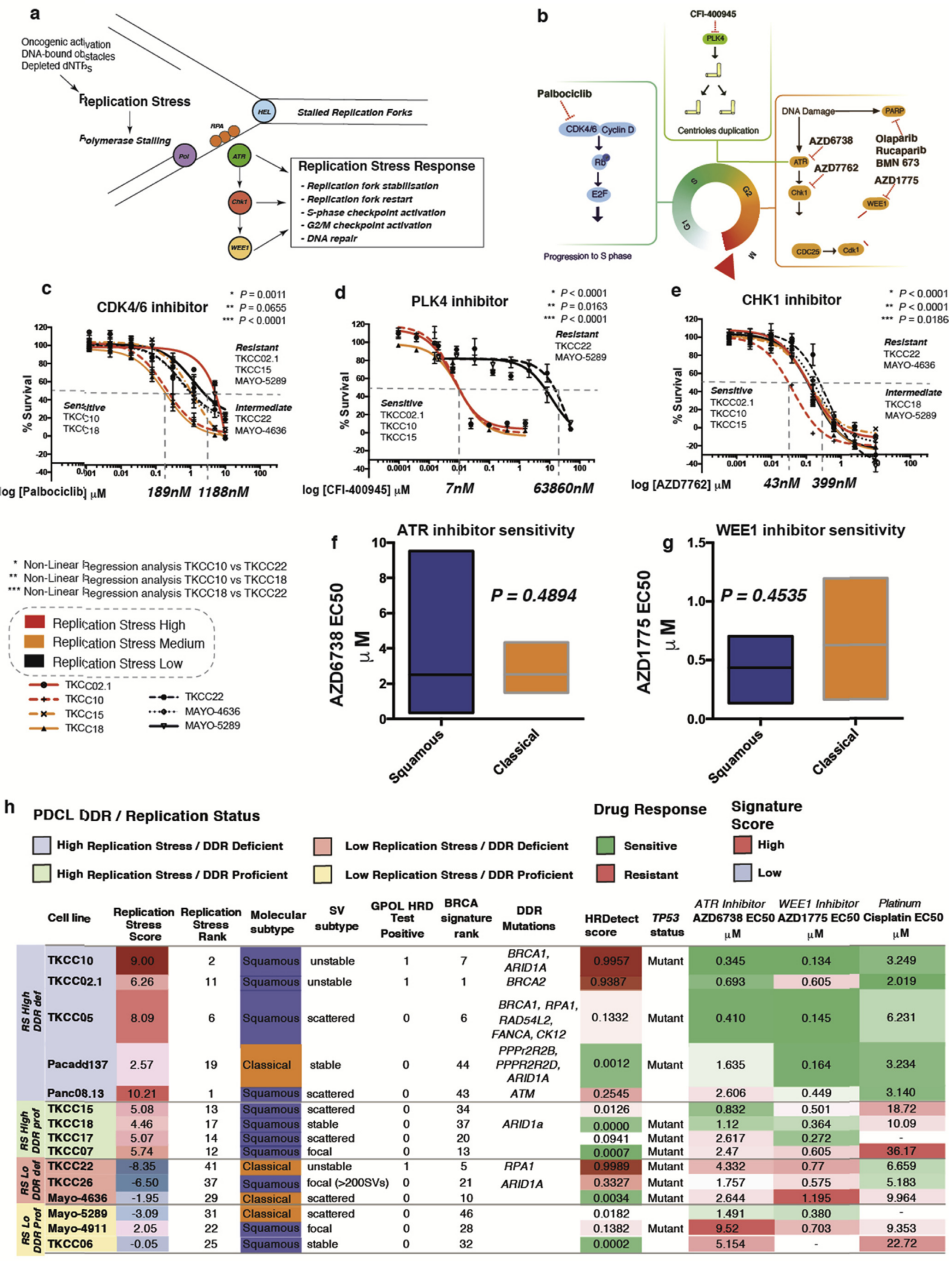
Targeting replication stress in PC PDCLs. (*A*) Replication stress can be induced by multiple factors, including oncogenic activation (KRAS, MYC) and chemotherapeutics (eg, platinum). This results in stalled replication forks when DNA polymerases (Pol) are separated from DNA helicase (HEL). This results in the coating of single-strand DNA by replication protein A (RPA), which results in ATR activation. This, in turn, generates the replication stress response via CHK1 and WEE1, resulting in checkpoint activation and DNA repair. This safeguards the integrity of the genome by preventing entry into mitosis with incompletely replicated genomes. (*B*) Agents currently in clinical trials or approved for use in other cancer types that target cell cycle checkpoints. Cell viability curves for agents inhibiting (*C*) CDK4/6 (palbociclib), (*D*) PLK4 (CDI-400945), and (*E*) CHK1 (AZD7762). PDCLs were classified by replication stress signature score as high (*red*), medium (*orange*), and low (*black*). (*F*) Differences in sensitivity to ATR inhibitor (AZD6738) in squamous and classical cell lines. (*G*) Differences in sensitivity to WEE1 inhibitor (AZD1775) in squamous and classical cell lines. (*H*) Response to DNA damaging agents and agents targeting cell cycle checkpoint. Colored heatmap reflects replication stress signature score and relative HRDetect score (*red* indicates high; *blue* indicates low) and drug sensitivity (*green* indicates most sensitive; *red* indicates resistant). In general, PDCLs with high replication stress are more sensitive to ATR and WEE1 inhibition, irrespective of DDR status. In general, platinum sensitivity is dependent on DDR status, irrespective of replication stress signature score. dNTP, deoxynucleotide triphosphate.

These results suggest that DDR deficiency, as defined by these putative biomarkers, have potential clinical utility in predicting response to platinum treatment. Importantly, this included both somatic and germline mutations, suggesting that therapeutic sensitivity extends beyond germline *BRCA1* and *2* mutations (Figure 2 and Supplementary Table 12). The most robust predictors appear to be biallelic loss-of-function mutations in *BRCA1* or *BRCA2*, or the presence of a genomic scar indicating loss of HR, such as high SVs (>200) and a positive GPOL HRD test. The COSMIC BRCA mutational signature appears to be a poor predictor of platinum response (Supplementary Figure 3).

### Replication Stress Is a Feature of the Squamous Subtype of Pancreatic Cancer

**Figure 3:**
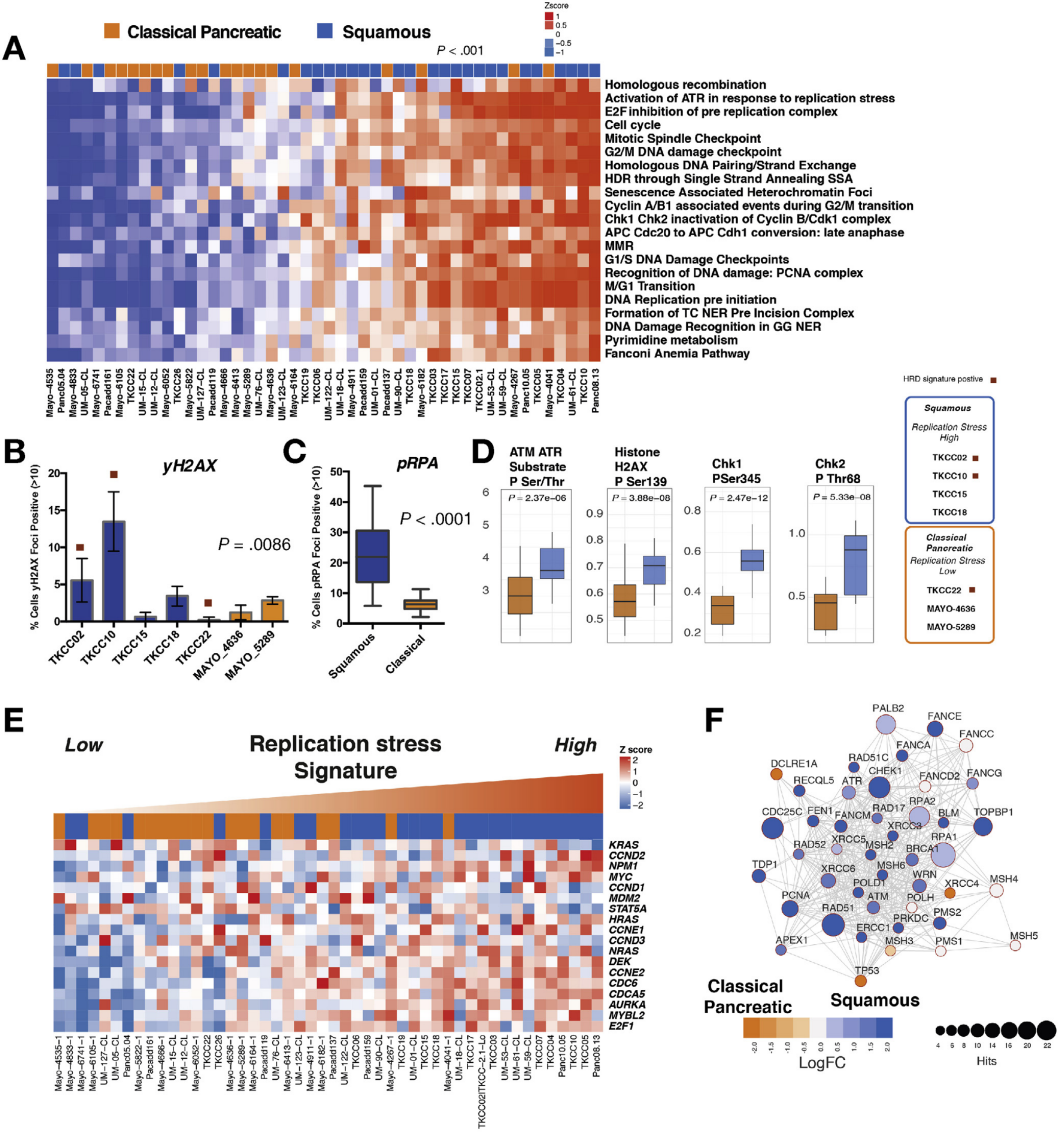
Replication stress in PDCLs of PC. (*A*) Heatmap of pathways and molecular processes (GO terms) involved in DNA maintenance and cell cycle regulation activated in replication stress and DNA damage response. PDCLs are ranked from right to left based on the decreasing novel transcriptomic signature score of replication stress, and molecular subtype is indicated in the top bar showing the association between activation of replication stress and the squamous subtype (*P* < .001, chi-square test, low vs high). (*B*, *C*) Immunofluorescent quantifications of (*B*) γH2AX and (*C*) pRPA at normal conditions are elevated in the squamous (*blue*) but not the classical pancreatic (*orange*) PDCLs. (*D*) Proteomic analysis using RPPA of a panel of PDCLs showed that replication stress response proteins are differentially activated in the squamous subtype. (*E*) Heatmap showing oncogene expression in PDCLs ranked from right to left by replication stress signature. Squamous PDCLs are enriched for oncogene activation and replication stress. (*F*) siRNA screening showing transcriptome functional interaction subnetwork, showing preferential dependencies of cell cycle control and DNA maintenance genes in the squamous subtype. Different node colors represent dependencies in different molecular subtypes, and the size of each node is relative to the number of siRNA hits. FC, fold change.

Replication stress has been described to be closely related to DDR deficiency, and activation of cell cycle genes is enriched in the squamous subtype. As a consequence, we investigated targeting of replication stress as a novel therapeutic strategy. We found significant subtype differences in the expression of genes controlling cell cycle, including the G2/M checkpoint in both PDCLs and bulk tumor PC (Figure 1)*.* Expression of *WEE1* (*P* = .006), *CDK6* (*P* = .02), and *CDK7* (*P* < .001) was enriched in the squamous subtype in both PDCLs and bulk tumor (Figure 1*A*). We then used a combination of DNA maintenance, replication, and cell cycle regulation network–related transcriptional profiles from GO and pathway enrichment analysis to define replication stress using mechanisms associated with DNA replication (ATR activation, chromosomal maintenance, E2F transcriptional pathways, HR, Fanconi anemia, base-excision repair, p53 signaling, endoplasmic reticulum stress, and RNA processing). This resolved into a transcriptomic signature (termed the *replication stress signature*), which was applied as a hypothetical biomarker of replication stress (Figure 3 and Supplementary Table 14).

**Supplementary Figure 4:**
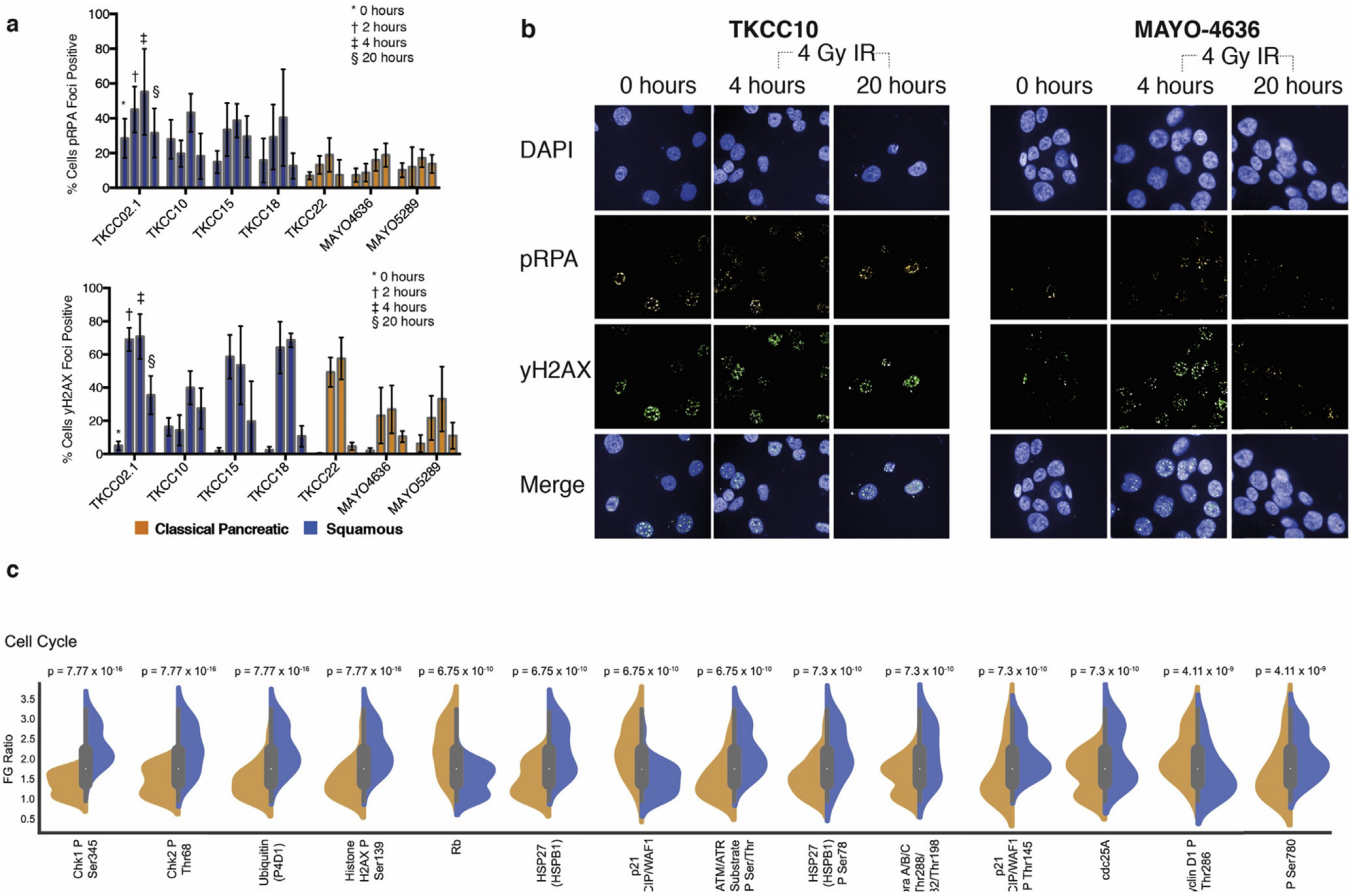
Replication stress in PC PDCLs. (*A*) Immunofluorescent quantification of pRPA and yH2AX after 4 Gy of ionizing radiation (IR) in classical pancreatic (*orange*) and squamous (*blue*) PDCLs. pRPA- and γH2AX-positive cells are defined as cells with >10 foci of pRPA and γH2AX per cell. (*B*) Immunofluorescent images of TKCC10 (squamous) and Mayo-4636 (classical pancreatic) PDCLs at normal and at 4 and 20 hours after 4 Gy IR. (*C*) Violin plots of RPPA analysis showing differential expression of (phospho-)proteins in, cell cycle, and DNA damage pathways between subtypes. Kruskal-Wallis test was used for violin plot *P* values. FG, femtogram.

PDCLs with high replication stress were more likely to be of the squamous subtype (*P* < .001) (Figure 3) and had significantly higher levels of pRPA at rest (a surrogate marker of single-stranded DNA break accumulation that infers replication stress) (*P* < .0001) (Figure 3). PDCLs with high replication stress and concurrent HRD had a greater proportion of γH2AX-positive cells at rest (Supplementary tables 15 and 16) (a marker of double-stranded DNA breaks) (*P* = .0086) and had persistently high levels of pRPA- and γH2AX-positive cells at 20 hours after ionizing radiation, when pRPA and γH2AX should have returned to normal levels in cells with no replication defects and competent at repairing this level of DNA damage (Supplementary Figure 4). RPPAs inferred functional consequences with differential phosphorylation and activation of key effectors of DDR and cell cycle progression between the classical and squamous subtypes (CHK1, CHK2, Rb, p21^CIP1^/^WAF1^, ATM/ATR substrates, cyclin D1, histone H2AX) (Figure 3, Supplementary Figure 4, and Supplementary Table 15). The squamous subtype is also enriched for the activation and transcription of oncogenes including *MYC* and *CCNE* (Figure 3). Oncogene activation is known to cause replication stress secondary to genomic instability, leading to activation of cell cycle checkpoint regulatory proteins involved in the replication stress response, such as ATR, WEE1, and CHK1^25^^,^^40^ (Supplementary Figure 3).

An siRNA screening targeting genes controlling DNA damage repair and replication showed a functional dependency on DDR proteins, including ATM, ATR, and CHK1 in squamous PDCLs (Figure 3*F*, Supplementary Figure 2, and Supplementary Table 17). This is in keeping with the results from the immunofluorescent and RPPA analyses suggesting higher baseline levels of proteins associated with replication stress in the squamous PDCLs and a subsequent dependency on these proteins and cell cycle checkpoints for maintaining genomic integrity and cell survival.

Differential expression of genes regulating the G2/M checkpoint in PDCLs and bulk tumors (such as *WEE1* and *CHEK1*) and the dependence on ATR activation in response to replication stress (Figure 3*A*) suggest that selective inhibition of these mechanisms may confer efficacy in tumors with high replication stress.

### Replication Stress Is Associated With Sensitivity to Cell Cycle Checkpoint Inhibitors

**Figure 4:**
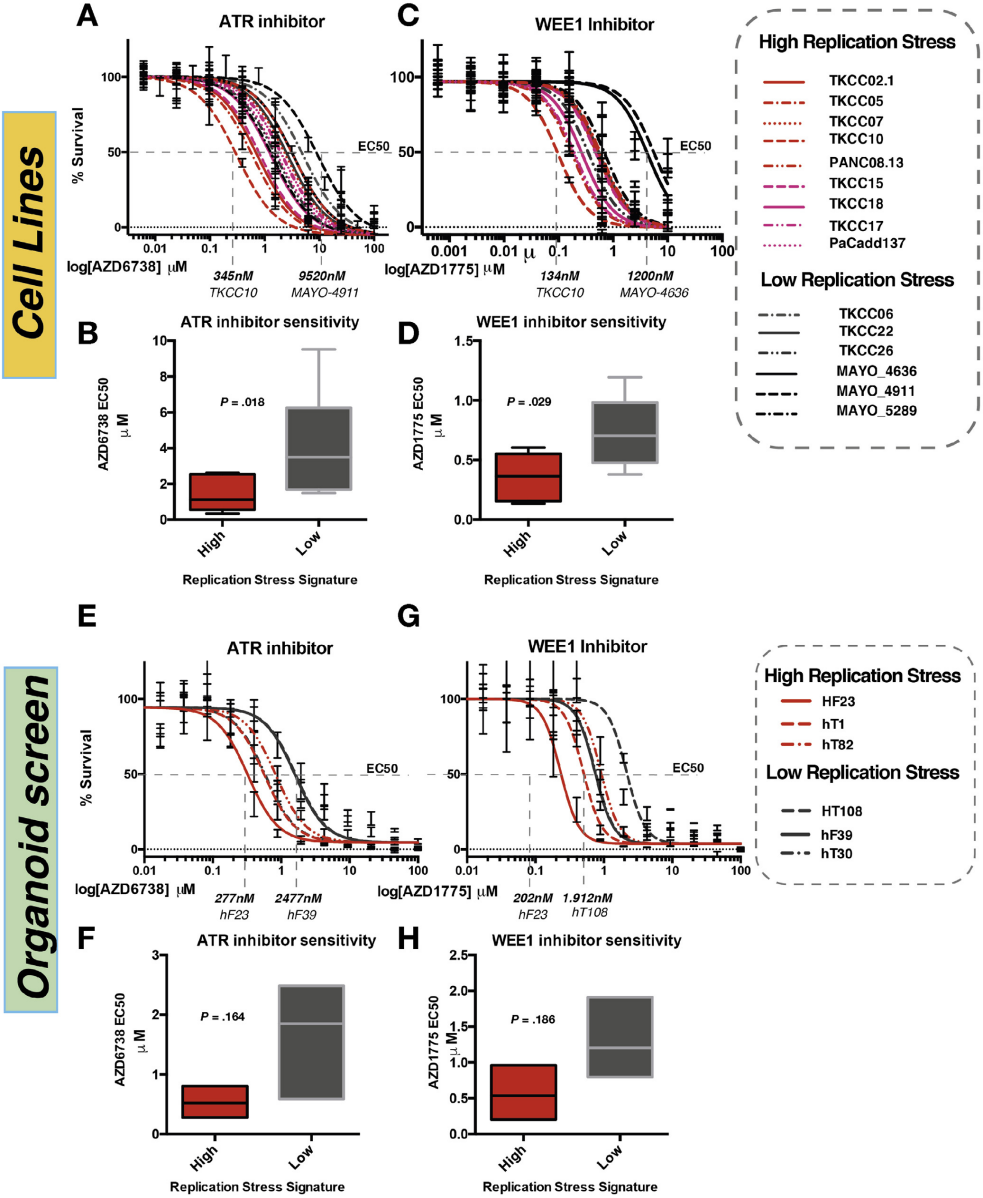
Targeting replication stress in PC. Dose response curves (EC_50_ shift) for (*A*) ATR and (*B*) WEE1 inhibitors calculated by using MTS assay in PDCLs after 72 hours of drug treatment. (*C*, *D*) Mean relative EC_50_ for PDCLs stratified by replication stress score. Patient-derived organoid drug screening dose response curves (EC_50_ shift) for (*E*) ATR and (*G*) WEE1 inhibitors calculated by using MTS assay after 72 hours of drug treatment. (*F*, *H*) Mean relative EC_50_ for PDCLs stratified by replication stress score. Each boxplot represents mean EC_50_, and box and whiskers represent minimum and maximum EC_50_ with 95% confidence interval. *P* calculated by using Mann-Whitney test between mean EC_50_ in each group.

Novel agents currently in early-phase clinical trial targeting the cell cycle were used to generate a therapeutic testing strategy (Supplementary Figure 3*B*).^41^, ^42^, ^43^, ^44^, ^45^ Based on these data, in vitro sensitivity was assessed by using cell viability assays after a selection of PDCLs were treated with increasing doses of inhibitors of CHK1 (AZD7762), CDK4/6 (palbociclib), and PLK4 (CFI-400945), showing differential sensitivity (Supplementary Figure 3 and Supplementary Table 18). Based on promising early clinical trial results in other cancer types,^44^, ^45^, ^46^, ^47^, ^48^, ^49^ more extensive testing using inhibitors of ATR (AZD6738) and WEE1 (AZD1775) was performed on 15 PDCLs defined as high and low replication stress based on the replication stress signature score (Figure 3*A* and Supplementary Figure 3). This showed that PDCLs with high replication stress were more sensitive to both ATR and WEE1 inhibition (Figure 4*A*–*D*). To validate these findings, we used a panel of human PC-derived organoids,^32^ which are deemed as the current criterion standard 3-dimensional model for pharmacotyping (Supplementary Tables 19 and 20). As seen in the PDCLs, organoids within the top quintile of high replication stress predicted response to both ATR and WEE1 inhibition with sensitivity to both agents in all organoids classified as high replication stress (Figure 4*E*–*H*). Importantly, these responses were independent of DDR status or molecular subtype, with responses seen in only high replication stress PDCLs (Supplementary Figure 3), suggesting that high replication stress signature is a more reliable biomarker of ATR inhibitor or WEE1 inhibitor response than squamous subtype or DDR deficiency.

### Replication Stress Is Independent of DNA Damage Response Deficiency in Pancreatic Cancer

**Figure 5:**
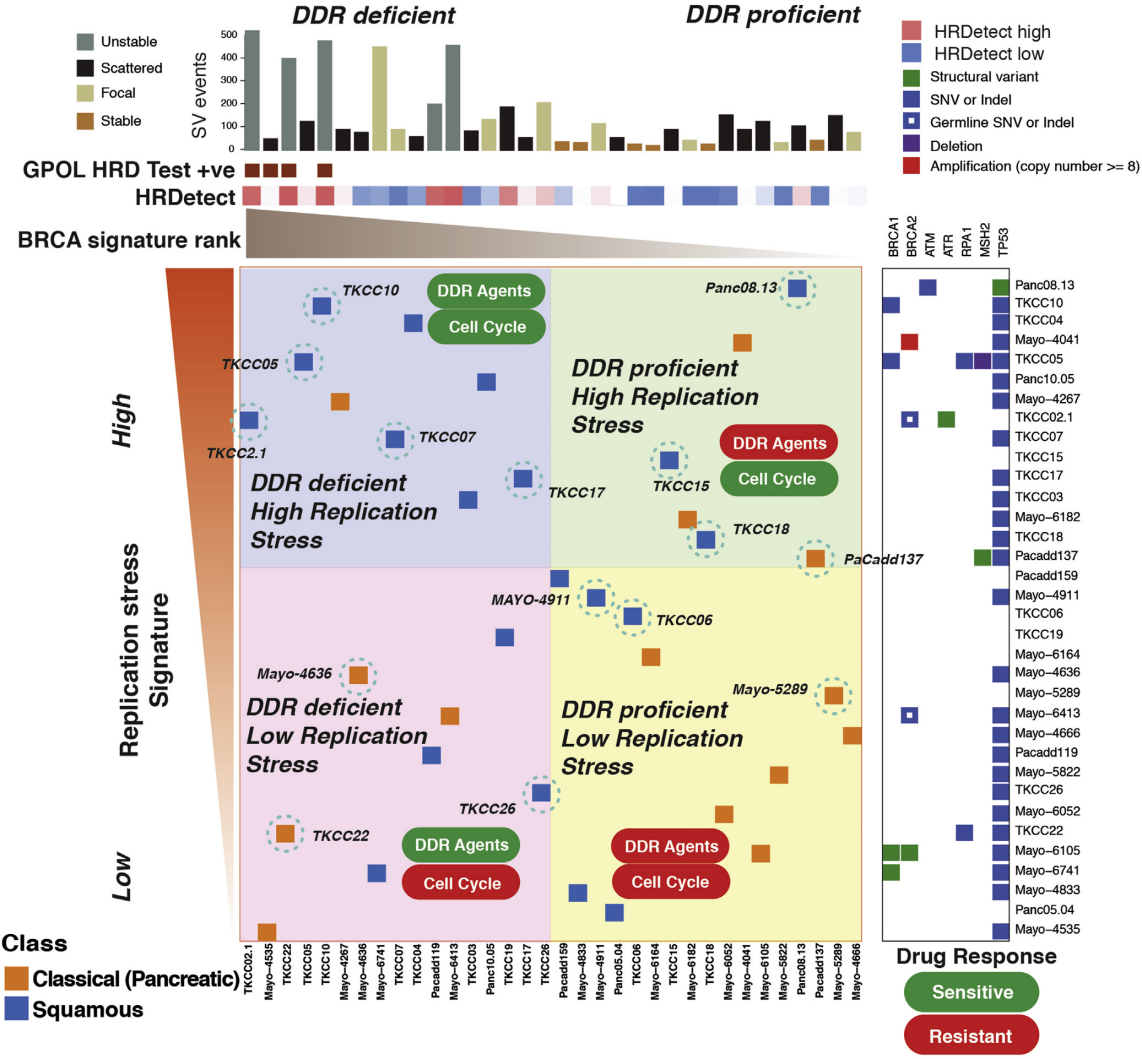
Relationship between DDR deficiency, replication stress, and therapeutic response in PDCLs of PC. PDCLs are ranked based on a novel transcriptomic signature of replication stress (*y*-axis) and a composite genomic readout of DDR deficiency (*x*-axis). DDR deficiency is a hierarchical score that incorporates the COSMIC BRCA mutational signature (signature 3), the number of structural variants distributed across the genome, and the GPOL HRD test associated with BRCA deficiency. Relative HRDetect score is indicated by colored scale. The combination of high/low states of each characteristic results in 4 groups. Squamous subtype PDCLs (*blue squares*) are associated with high replication stress (*P* = .007, chi-square test). PDCLs tested are identified and encircled in blue. DDR deficiency and the replication stress signature predicted differential therapeutic response.

To investigate the relationship of replication stress and DDR deficiency and the alignment of these therapeutic segments, a comparison was performed with the PDCL cohort. By using the described biomarkers of DDR deficiency and replication stress, a 2-by-2 grid was constructed to compare replication stress ranking and DDR deficiency (Figure 5). This showed that signatures of DDR deficiency and replication stress are largely independent of each other, yet high replication stress is enriched in the squamous subtype (*P* = .007) (Figure 5). Therapeutic response data were overlapped based on previously described experiments by using ATR/WEE1 inhibitors and platinum, generating biomarker hypotheses for therapeutic responsiveness (Figure 5). PDCLs that are DDR deficient with high replication stress respond to both DDR targeting agents (eg, platinum and PARP inhibitors) and cell cycle checkpoint inhibitors (eg, ATR and WEE1 inhibitors), DDR-deficient PDCLs with low replication stress respond to DDR agents only, DDR-proficient PDCLs with high replication stress respond to cell cycle checkpoint inhibitors only, and DDR-proficient PDCLs with low replication stress respond to neither class of agent (Figure 5).

### Potential Clinical Utility of the Replication Stress Signature

**Supplementary Figure 5:**
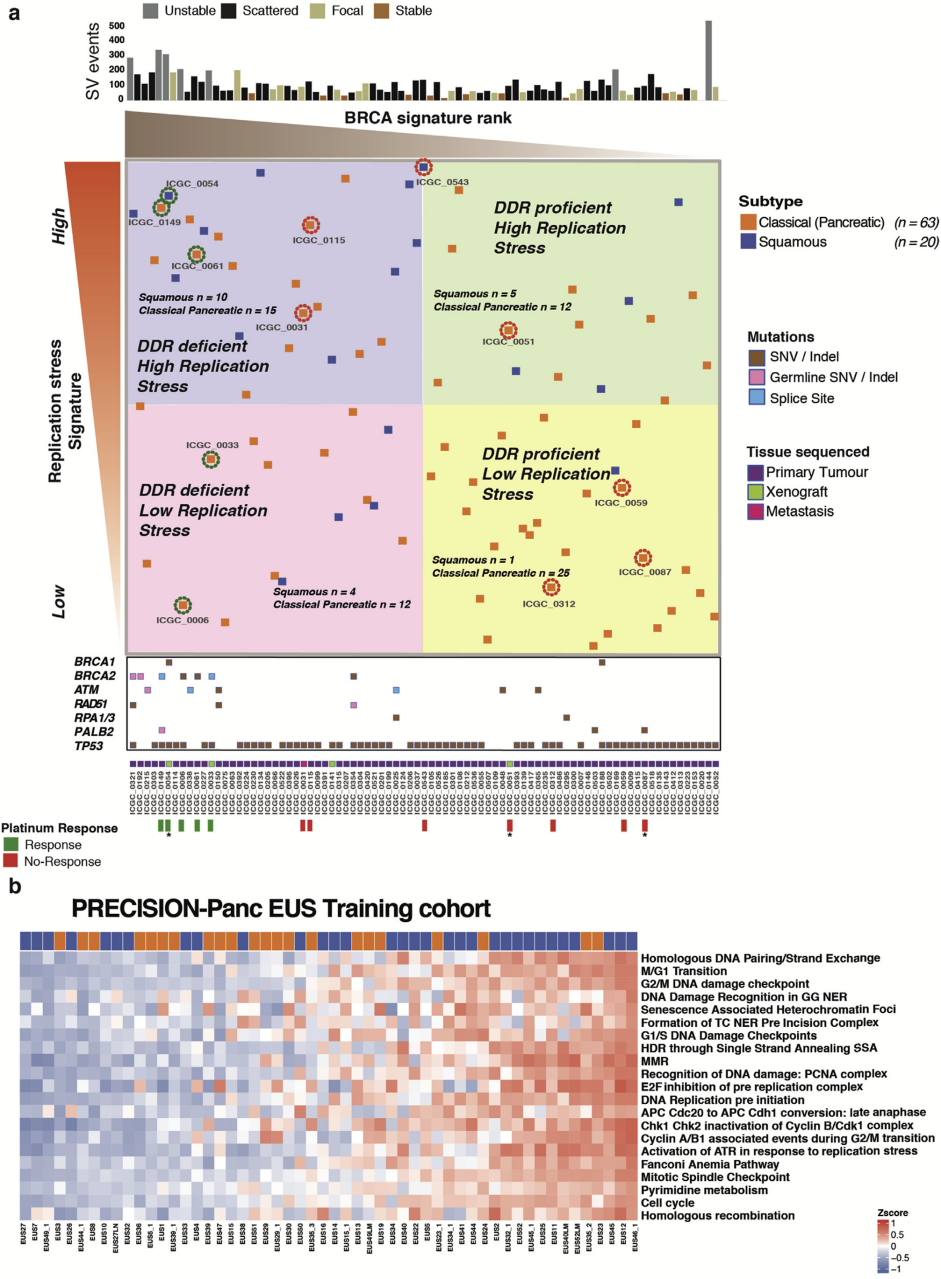
Targeting replication stress and DDR deficiency in clinical cohorts of PC. (*A*) Bulk tumor samples from the ICGC PC cohort that have undergone both whole-genome sequencing and RNAseq are ranked from left to right based on the COSMIC BRCA mutational signature as a scale of DDR deficiency (*x*-axis) and top to bottom by the novel transcriptomic signature of replication stress (*y*-axis). HR pathway gene mutations and source of tissue sequenced are marked along the *x*-axis. Platinum response is marked along *x*-axis, and the related patient is encircled at individual points, where green represents response, and red indicates resistance. An asterisk indicates PDX response data. Relevant molecular subtype frequency (squamous vs classical pancreatic) is indicated for each quadrant, showing that squamous PC was associated with high-ranking replication stress score (15 out of 41 vs 5 out of 42) (*P* = .009, chi-square test). (*B*) The replication stress signature in the Precision-Panc endoscopic ultrasonography fine-needle biopsy training cohort, showing its clinical utility in the advanced disease setting (34% of cohort was locally advanced, and 37% was metastatic). The top-ranking quartile of replication stress signature scored as high showed that 50% of squamous tumors were within this group, compared to only 21% of the classical pancreatic tumors (*P* = .027, chi-square test). EUS, endoscopic ultrasonography; SNV, single-nucleotide variant.

To assess the potential clinical validity and utility of these preclinical data, the relationship between the replication stress signature score and molecular subtypes in bulk tumor samples was assessed by using published transcriptomic data sets of PC.^5^^,^^9^ This included whole transcriptome sequencing sets acquired through the ICGC totaling 94 patients with primary resected PC (Supplementary Figure 5). This recapitulated the association between squamous molecular subtype and high replication stress (*P* = .006) (Supplementary Figure 5), with 50% of squamous tumors in the top quartile of tumors ranked by the replication stress score.

**Supplementary Figure 6:**
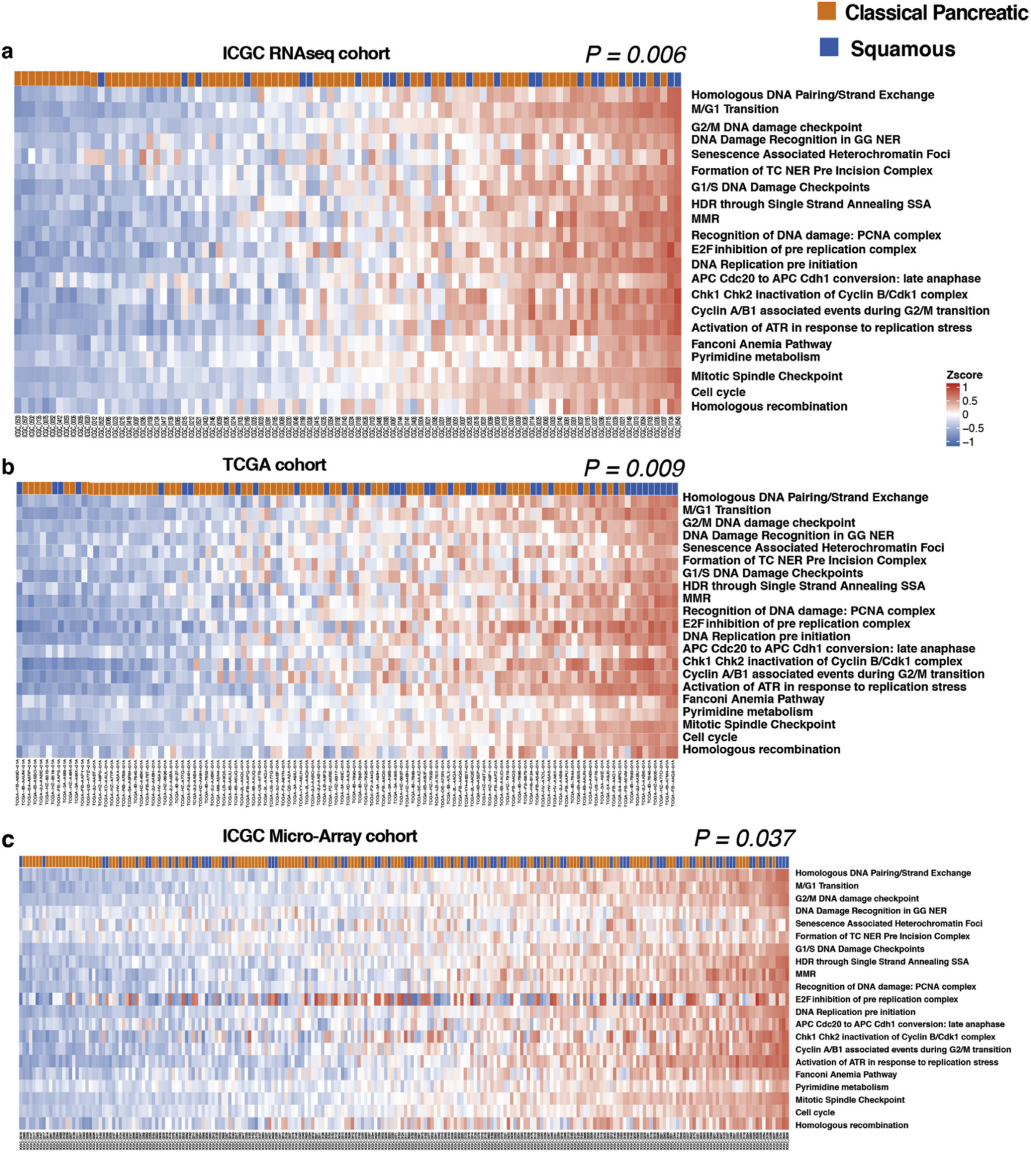
Replication stress signature in published bulk tumor cohorts of PC. The association between molecular subtype and replication stress in the (*A*) ICGC RNAseq (n = 94), (*B*) TCGA (n = 112), and (*C*) ICGC microarray (n = 232) cohorts. In the ICGC (*P* = .006), TCGA (*P* = .009), and ICGC microarray cohorts (*P* = .037), high replication stress was significantly enriched for the squamous subtype. High replication stress was defined as the top-ranking quartile in this cohort; *P* was calculated using the chi-square test.

The replication stress signature was then applied to The Cancer Genome Atlas^9^ high epithelial cellularity set (ABSOLUTE purity ≥0.2), and the ICGC microarray transcriptomic data sets (Supplementary Figure 6). Again, the top-ranking quartile of replication stress signature was significantly enriched with squamous subtype PC (The Cancer Genome Atlas set, *P* = .009; microarray set, *P* = .037) (Supplementary Figure 6). We then examined the potential clinical utility of the replication stress signature in biopsy material acquired through the Precision-Panc endoscopic ultrasound fine-needle biopsy training cohort (n = 54), recruited and collected during the development of the Precision-Panc (Supplementary Figure 5*B*).^50^ As in the other cohorts, this showed enrichment of the squamous subtype with high replication stress (*P* = .027) and provides proof-of-principle clinical validity that the signature can be generated from fine-needle biopsy material and used as a putative biomarker in the clinical setting.

## Discussion

Identifying responsive patient subgroups is crucial to therapeutic development and improving outcomes for PC. Genomic sequencing studies and the development of novel therapeutic agents has made DDR mechanisms one of the most attractive therapeutic opportunities in PC.^4^^,^^8^ Using surrogate markers of DDR deficiency (GPOL HRD test, structural variation, the COSMIC BRCA mutational signature, and mutations in HR pathway genes) we show that DDR-deficient PCs respond preferentially to both platinum and PARP inhibitors in PDCLs (n = 15) and long-lasting complete and near-complete responses in a DDR-deficient PDX model with single-agent PARP inhibition with olaparib. This was as effective as cisplatin monotherapy or combination treatment with cisplatin and Olaparib, suggesting that in appropriately selected patients, PARP inhibitor monotherapy can potentially induce clinically relevant responses similar to platinum. This provides potential therapeutic options for patients with poor performance status or after intolerance or acquired resistance to platinum has developed.^36^ Predicting platinum response is more complex than using point mutations in DDR genes alone. In keeping with other studies,^51^ biallelic loss-of-function mutations in HR genes, structural variation signatures, including >200 SVs,^4^ and the GPOL HRD test appear to be robust, but they require testing in clinical settings. However, the COSMIC BRCA mutational signature is a poor predictor of platinum response in isolation. Selecting robust biomarkers for platinum response is crucial for clinical testing. The GPOL HRD test in conjunction with mutations in DDR genes has been selected as the biomarker of platinum response to investigate in PRIMUS-001 (ISRCTN75002153) and PRIMUS-002 (ISRCTN34129115), 2 phase 2/3 clinical trials on the Precision-Panc platform.

We define a novel replication stress signature which is associated with the squamous subtype in PDCLs and bulk tumors from multiple PC cohorts (n = 383 patients). Elevated replication stress, as defined by this signature, is associated with functional deficiencies in DNA replication, leading to a therapeutic vulnerability to novel agents as demonstrated by cell viability assays, organoid drug screenings, and siRNA functional screening. This molecular feature is independent of DDR status, platinum response, or molecular subtype. This suggests that molecular signatures, such as the replication stress signature, can be used as biomarkers for predicting response to ATR or WEE1 inhibitors and offer patients with DNA replication defects alternative therapeutic options to the standard-of-care platinum chemotherapy. Tumors that are DDR deficient can be targeted with platinum-based therapy or, in the context of a patient with reduced performance status or as the second line, PARP inhibitors. Patients with high replication stress can be targeted with ATR or WEE1 inhibitors, which can be combined with PARP inhibitors or platinum if concurrent DDR deficiency exists or after platinum resistance develops. This hypothesis will be tested in the PRIMUS-004 (ISRCTN16004234) clinical trial on the Precision-Panc platform, with the secondary endpoint of replication stress signature as a biomarker of response.

In summary, we developed and performed preclinical testing on novel biomarkers of DDR deficiency and replication stress that have potential clinical utility. Well-designed precision oncology platforms, such as Precision-Panc (precisionpanc.org), will enable biomarker-driven clinical testing and allow refinement of biomarkers predicting meaningful responses and potential translation into clinical practice.

## Supplementary Materials and Methods

### Australian Pancreatic Cancer Genome Initiative

Sydney South West Area Health Service Human Research Ethics Committee, western zone (protocol number 2006/54); Sydney Local Health District Human Research Ethics Committee (X11-0220); Northern Sydney Central Coast Health Harbour Human Research Ethics Committee (0612-251M); Royal Adelaide Hospital Human Research Ethics Committee (091107a); Metro South Human Research Ethics Committee (09/ QPAH/220); South Metropolitan Area Health Service Human Research Ethics Committee (09/324); Southern Adelaide Health Service/Flinders University Human Research Ethics Committee (167/10); Sydney West Area Health Service Human Research Ethics Committee (Westmead campus) (HREC2002/3/4.19); The University of Queensland Medical Research Ethics Committee (2009000745); Greenslopes Private Hospital Ethics Committee (09/34); North Shore Private Hospital Ethics Committee. Johns Hopkins medical institutions: Johns Hopkins Medicine institutional review board (NA00026689). Ethikkommission an der Technischen Universität Dresden (approval numbers EK30412207 and EK357112012). University of Michigan institutional review board (HUM00025339). Mayo Clinic institutional review board (no. 66-06)

### PRECISION-Panc Endoscopic Ultrasound Training Cohort

Ethical approval was obtained for collecting additional research biopsy samples from patients undergoing endoscopic ultrasound-guided biopsies for investigation of possible PC (ethical approval number: 17/WS/0085). Fully informed consent was obtained for all patients from whom additional biopsy samples were taken. Samples were anonymized at the point of collection, with only the PhD candidate being able to identify patients from research samples.

### Whole-Genome Library Preparation

Whole-genome libraries were generated by using either the Illumina TruSeq DNA LT sample preparation kit (part nos. FC-121–2001 and FC-121–2001) or the Illumina TruSeq DNA PCR-Free LT sample preparation kit (part nos. FC-121–3001 and FC-121–3002) according to the manufacturer’s protocols with some modifications (Illumina, part nos. 15026486 Rev. C July 2012 and 15036187 Rev. A January 2013 for the 2 different kits, respectively). For the TruSeq DNA LT sample preparation kit, 1 μg of genomic DNA was used as input for fragmentation to approximately 300 base pairs (bp), followed by an Solid Phase Reversible Immobilization (SPRI)-bead cleanup with the AxyPrep Mag PCR Clean-Up kit (Corning, part no. MAG-PCR-CL-250). After end repair, 3′ adenylation, and adaptor ligation, the libraries were size-selected by using a double SPRI-bead method to obtain libraries with an insert size of approximately 300 bp. The size-selected libraries were subjected to 8 cycles of polymerase chain reaction (PCR) to produce the final whole-genome libraries ready for sequencing. For the TruSeq DNA PCR-Free LT sample preparation kit, 1 μg of genomic DNA was used as input for fragmentation to approximately 350 bp, followed by an end-repair step and then a size selection using the double SPRI-bead method to obtain libraries with an insert size of approximately 350 bp. The size-selected libraries then underwent 3′ adenylation and adaptor ligation to produce final whole genome libraries ready for sequencing. Before sequencing, whole-genome libraries were qualified via the Agilent (Santa Clara, CA) BioAnalyzer 2100 with the High Sensitivity DNA Kit (Agilent, part no. 5067-4626). Quantification of libraries for clustering was performed using the KAPA Library Quantification Kit–Illumina/ Universal (KAPA Biosystems, part no. KK4824) in combination with the Life Technologies Viia 7 real-time PCR instrument.

### RNA-Sequencing Library Generation and Sequencing

RNA-seq libraries were generated by using TruSeq Stranded Total RNA (part no. 15031048 Rev. D April 2013) kits on a PerkinElmer Sciclone G3 NGS Workstation (product no. SG3- 31020-0300). Ribosomal depletion step was performed on 1 μg of total RNA by using Ribo-Zero Gold before a heat fragmentation step aimed at producing libraries with an insert size between 120 and 200 bp. Complementary DNA was then synthesized from the enriched and fragmented RNA by using Invitrogen (San Diego, CA) SuperScript II Reverse Transcriptase (catalog no. 18064) and random primers. The resulting cDNA was further converted into double-stranded DNA in the presence of deoxyuridine triphosphate to prevent subsequent amplification of the second strand and thus maintain the strandedness of the library. After 3′ adenylation and adaptor ligation, libraries were subjected to 15 cycles of PCR to produce RNAseq libraries ready for sequencing. Before sequencing, exome and RNAseq libraries were qualified and quantified via the Caliper LabChip GX (part no. 122000; East Lyme, CT) instrument using the DNA High Sensitivity Reagent kit (product no. CLS760672). Quantification of libraries for clustering was performed with the KAPA Library Quantification Kits for Illumina sequencing platforms (kit code KK4824) in combination with Life Technologies Viia 7 real-time PCR instrument.

### Weighted Gene Coexpression Network Analysis

Weighted gene coexpression network analysis (WGCNA) was used to generate a transcriptional network from rlog-normalized RNAseq data.^1^ Briefly, WGCNA clusters genes into network modules by using a TOM. The TOM is a highly robust measure of network interconnectedness and essentially provides a measure of the connection strength between 2 adjacent genes and all other genes in a network. Genes are clustered by using 1 – TOM as the distance measure, and gene modules are defined as branches of the resulting cluster tree using a dynamic branch-cutting algorithm.

The module eigengene is used as a measure of module expression in a given sample and is defined as the first principal component of a module. To relate sample traits of interest to gene modules, sample traits were correlated to module eigengenes, and significance was determined by a Student asymptotic *P* value for the given correlations. To relate gene modules to PDCL subtypes, module eigengenes were stratified by subtype, and subtype significance was determined by the Kruskal-Wallis test.

Module preservation as implemented in WGCNA detects the conservation of gene pairs between 2 networks (eg, PDCL and bulk). Two composite measures were used to assess module preservation, namely, median rank and *Z*_summary_. Median rank was used to identify module preservation, and *Z*_summary_ was used to assess the significance of module preservation via permutation testing. Permutation was performed 200 times; modules with a *Z*_summary_ score of >10 indicate preservation, 2–10 indicates weak to moderate preservation, and <2 indicates no preservation in the permutations.

Reverse Phase Protein Array. Samples were lysed in RIPA lysis buffer (50mM Tris-HCL at pH7.4, 150mM Sodium Chloride, 5mM EGTA, 0.1% SDS, 1% NP40, 1% Deoxycholate, supplemented protease and phosphatase inhibitor tablets; Roche Applied Science Cat. #: 05056489001 and 04906837001) for 30 min on ice and cleared by centrifugation at 14K for 15 min at 4°C. Protein concentration was determined using a Bradford assay (Sigma) and all samples were normalized to 2mg/ml. 4x SDS sample buffer (40% Glycerol, 8% SDS, 0.25M Tris-HCL, pH 6.8. 1/10th vol/vol 2-mercaptoethanol) was added to each sample, followed by incubation at 80°C for 5 mins. Serial dilutions (1, 0.5, 0.25, 0.125) were then prepared by diluting samples in PBS. Samples were printed onto Avid nitrocellulose coated glass slides (Grace Biolabs) using an Aushon 2470 microarrayer (Aushon Biosystems), with 2 technical replicates per sample. Slides were processed as follows: 4 x 15 min washes with dH20, incubated with antigen retrieval agent (Reblot strong, Millipore) for 15 min, 3 x 5 min washes with PBST, incubated with superblock TBST (ThermoFisher Scientific) for 10 min, 3 x 5 min washes with TBST, incubated with primary antibodies (all 1:200) diluted in superblock TBST for 60 mins, 3 x 5 min washes with TBST, blocked with superblock TBST for 10 mins, 3 x 5 min washes with TBST, incubated with anti-rabbit dylight 800 secondary Ab (1:2000 in superblock TBST)(Cell Signalling Technologies) for 30 mins, 3 x 5 min washes with TBST, 1 x 5 min wash with dH20, slides spun at 2000rpm for 5mins and allowed to air dry in the dark. An additional slide was stained with FAST Green FCF for normalization against total protein: 3 x 5min washes with dH20, incubated for 15 mins in 1% NaOH, slides rinsed 20 x in dH20, incubated for 10 min in dH20, incubated in de-stain (30% methanol, 7% glacial acetic acid, 63% dH20) for 15 min, incubated for 3 mins in FAST green staining solution (0.0025%w/v FAST green in de-stain), rinsed 20 x in dH20, incubated for 15 mins in de-stain solution, rinsed 20 x in dH20, spun at 2000rpm for 5 mins and allowed to air dry in the dark. All steps were performed at room temperature with agitation. Slides were visualized using an Innopsys 710AL infra-red microarray scanner and signals quantified using MAPIX microarray image analysis software (Innopsys). Non-specific signals were determined by omitting the primary antibody incubation step. All signals were within the linear range of detection with an R2 value >0.9. Final output is the median value for each dilution series as fentogram, background subtracted and normalized for protein loading.

### Small Interfering RNA Screening

Before siRNA screening, the optimal cell number per well and optimal reverse transfection reagents for each PDCL were identified by assessing transfection efficiency using 6 different transfection reagents (Dharmafect 1-4, RNAimax, Lipofectamine 2000; Invitrogen, ThermoFisher), based on the manufacturers’ instructions. Experimental conditions were selected that met the following criteria: (1) compared to a mock control (no lipid, no siRNA), the transfection of nonsilencing negative control siRNA caused no more than 20% cell inhibition; (2) compared to nonsilencing negative control siRNA, the transfection of PLK1–targeting siRNA caused more than 80% cell inhibition; and (3) cell confluency reached 70% within the range of 4–7 days.^2^ The last criterion allowed assays to be terminated while cells were in the growth phase. Once optimal conditions were established, each PDCL was reverse transfected in a 384-well plate format with a custom siGENOME siRNA library (Dharmacon, Lafayette, CO) designed to target 714 kinase-coding genes, 256 protein phosphatase-coding genes, 722 genes implicated in energy metabolism, 73 tumor suppressor genes, and 166 genes involved in the repair of DNA damage (see Supplementary Table 19 for a list of genes covered in the siRNA library). Each well in the 384-well plate arrayed library contained a SMARTpool of 4 distinct siRNA species targeting different sequences of the target transcript. Each plate was supplemented with nontargeting siCONTROL and siPLK1 siRNAs (Dharmacon). Cell viability was estimated 5 days after transfection by using a luminescent assay detecting cellular ATP levels (CellTitre-Glo, Promega). Luminescence values were processed by using the cellHTS2 R package.^3^ To evaluate the effect of each siRNA pool on cell viability, we log2 transformed the luminescence measurements and then centered these to the median value for each plate. The plate-centered data were scaled to the median absolute deviation of the library as a whole to produce robust *Z* scores. All screenings were performed in triplicate. Screenings judged to have poor dynamic range (*Z′* factor < 0)^4^ or poorly correlated replicates (*r* < 0.7) were excluded during an evaluation of screening quality. *Z* scores were adjusted using a quantile normalization.^5^

### Small Interfering RNA Screening Analysis

siRNA hits were identified by calculating the median absolute deviation of normalized *Z* scores for a given siRNA across all samples and identifying sample *Z* scores greater than or equal to 2 × the median absolute deviation. This analysis generated a seed matrix (n siRNA hits × m samples), which was used as the starting input for the random walk with restart algorithm as implemented by the R package dnet*.*^6^ This algorithm was used to identify functionally important subnetworks associated with cell viability from a curated protein-protein interaction network, STRING, version 10.^7^ Considering the complex nature of topologic features of human interactome data, we introduce a randomization-based test to evaluate the candidate interactors using 1000 topologically matched random networks. Candidate interactors that remain significant (ie, *P*_edge_ < .05) were identified, and a consensus subnetwork was constructed by collapsing all sample-specific results. The resulting network was visualized by using RedeR.^8^

### RNA-Sequencing Analysis

RNAseq read mapping was performed by using the bcbio-nextgen project RNAseq pipeline (https://bcbio-nextgen.readthedocs.org/en/latest/). Briefly, after quality control and adaptor trimming, reads were aligned to the GRCh37 genome build using STAR^9^ counts for known genes were generated by using the function featureCounts in the R/Bioconductor package Rsubread.^10^ The R/Bioconductor package DESeq2 was used to normalize count data between samples and to identify differentially expressed genes.^11^ Expression data were normalized using the rlog transform in the DESeq2 package, and these values were used for all downstream analyses.

#### Weighted Gene Coexpression Network Analysis

WGCNA was used to generate a transcriptional network from rlog-normalized RNAseq data.^1^ Briefly, WGCNA clusters genes into network modules using a TOM. The TOM is a highly robust measure of network interconnectedness and essentially provides a measure of the connection strength between 2 adjacent genes and all other genes in a network. Genes are clustered by using 1 – TOM as the distance measure and gene modules are defined as branches of the resulting cluster tree using a dynamic branch-cutting algorithm.

### Identification of Significant Subtype-Specific Changes in Pathways and/or Processes

The R package clipper^12^ was used to identify pathways and/or processes showing significant change between PDCL subtypes. clipper implements a 2-step empirical approach, using a statistical analysis of means and concentration matrices of graphs derived from pathway topologies, to identify signal paths having the greatest association with a specific phenotype.

### Pathway Analysis

Ontology and pathway enrichment analysis was performed by using the R package dnet and/or the ClueGO/CluePedia Cytoscape^13^^,^^14^ plugins as indicated. Visualization and/or generation of network diagrams was performed with either Cytoscape^15^ or the R package *RedeR.*^8^

### Replication Stress Signature Generation

To define subtype-specific signatures that can inform therapeutic development, differentially expressed genes were compared to genes associated with GO terms by using the R package dnet. Significantly expressed GO terms involved in DDR and cell cycle control were selected. Differential expression of each selected GO term was applied to each individual PDCL that underwent RNAseq. This, in turn, was used to generate a composite score by totaling the score for each selected GO term. The *sig.score* function from the R package genefu^16^ was used to calculate a specific signature score in a given sample by using the signatures generated for each pathway and/or process. This was termed the replication stress signature. Generation of signature score for bulk tumor samples followed the same methodology.

### Bulk Expression Sets and Immune Signature Scores

Bulk RNAseq expression data, subtype assignments, and immune signature scores were obtained from Bailey et al.^17^

### Gene Set Enrichment of Pancreatic Ductal Adenocarcinoma Subtypes

Gene set enrichment was performed by using the R package GSVA.^18^ Gene sets representing PDAC subtypes were generated as previously described.^17^

### Clustering and Subtype Assignment

The package ConsensusClusterPlus^9^ was used to classify PDCLs according to the expression signatures defined in Moffitt et al^20^ and Bailey et al.^7^ Gene sets representing PDAC subtypes were generated as previously described.

### Statistical Analysis

A Kruskal-Wallis test was applied to the indicated stratified scores to determine whether distributions were significantly different. Fisher exact tests were used to evaluate the association between dichotomous variables.

### Plot Generation

Heatmaps and oncoplots were generated using the R package ComplexHeatmap*.*^21^ Dot charts, density plots, and boxplots were generated using the R package ggpubr*.* Violin plots were generated with the Python package *Seaborn.* Biplot was generated by using the R package ggfortify.^22^ All other plots were generated using the R package ggplot2*.*^23^

### Patient-Derived Xenograft Therapeutic Testing

PDX of PDAC were generated and comprehensively characterized as part of the ICGC project.^24^, ^25^, ^26^ BALB/c nude mice were anesthetized, and a single PDX fragment was inserted subcutaneously into the right flank according to standard operating procedure. PDX models were grown to 150 mm^3^ (volume = [length^2^ × width]/2); at this point, each PDX was randomized to a different treatment regime. Responsive PDXs were treated once tumor size returned to 150 mm^3^, up to a maximum of 3 rounds. Resistant models were treated after a treatment break of 2 weeks in accordance with current clinical treatment regimes, up to a maximum of 2 rounds. Each experiment was terminated once tumor volume reached the end point (750 mm^3^), in accordance with home office animal welfare regulations.

Upon tumor threshold, mice were treated with either cisplatin, gemcitabine, olaparib, saline (control), or combinations of these (Supplementary Tables 3–4). As PDXs were reaching the threshold, treatment allocation was done on a random basis, with a random number generator. Each PDX was treated with the subsequent treatment regimen upon reaching the threshold depending on the random order generated for that round. This ensured no bias in selecting PDXs to treatments. Phosphate-buffered saline (control) was allocated during every second round of treatments.

Treatment cycles were completed after 4 weeks. If tumor response was observed, then further cycles were given if only tumor growth returned to the threshold (150 mm^3^). In cases in which no significant response was seen, a 2-week treatment break was given to all animals, and then a second cycle was restarted. The maximum number of cycles that each animal was exposed to was 3 cycles.
